# Cellulose Functionalization Using *N*-Heterocyclic-Based Leaving Group Chemistry

**DOI:** 10.3390/polym16010149

**Published:** 2024-01-03

**Authors:** Arvind Negi, Ali R. Tehrani-Bagha

**Affiliations:** Department of Bioproducts and Biosystems, School of Chemical Engineering, Aalto University, 02150 Espoo, Finland; arvind.negi@helsinki.fi

**Keywords:** cellulose functionalization, surface modification, cellulose chemistry, activators, leaving group chemistry, coloration

## Abstract

There has been continuous interest in developing novel activators that facilitate the functionalization of cellulosic materials. In this paper, we developed a strategy in which trisubstituted triazinium salts act as cellulose preactivators. As leaving groups, these triazinium salts utilize *N*-heterocycles (pyridine, imidazole, and nicotinic acid). Initially, we optimized the synthetic route for developing these novel cellulose preactivators (triazinium salts), whose structures were confirmed using NMR spectroscopy. The surface zeta potential of cellulose changed from a negative value to a positive one after preactivation due to the cationic nature of these preactivators. To enhance the scope of the study, we functionalized the cellulose-preactivated materials with a series of amine- or hydroxy-containing aliphatic and aromatic hydrocarbons, nucleophilic amino acids (cysteine), colorants (2-aminoanthraquinone and 2-amino-3-methyl-anthraquinone), and biopolymer (zein protein). The treated samples were analyzed using FTIR, time-gated Raman spectroscopy, and reflection spectroscopy, and the success of the functionalization process was validated. To widen the scope of such chemistries, we synthesized four reactive agents containing *N*-heterocyclic-based leaving groups (pyridine and nicotinic acid) and successfully functionalized cellulose with them in one step. The proposed single- and two-step functionalization approaches will provide opportunities for chemically linking various chemical compounds to cellulose for different applications.

## 1. Introduction

Reactive chemical auxiliaries are used to activate cellulose hydroxy groups for subsequent functionalization. Cellulose structure contains (1,4) linked *β*-D glucopyranosyl units that are molecularly arranged within the hierarchical structures of microfibrils. These intermolecular arrangements provide strong mechanical properties to cellulose materials [1,2]. With such materialistic properties and as a most naturally abundant polymer, cellulose is used as a substrate, precursor, and supporting material to develop various commercial products, such as paper [3] and synthetic cellulose fibers [4]. The hydrogen bond network and molecular packing impart high crystallinity in cellulosic materials, making them chemically less penetrable. Therefore, cellulose functionalization requires preactivation, either with the help of reactive chemicals (surface-modifying chemicals, such as epoxides, azides, and amines) [5,6,7,8] or by enforcing other energy-consuming processes (such as photoactivation, hydrothermal, thermomechanical) [9,10,11,12,13]. Due to the availability of a wide range of reactive chemical auxiliaries for cellulose material processing, various commercial-grade cellulose forms have been discovered, including nitrocellulose, sulpho-cellulose, cellulose acetate, and cellulose phosphate. These chemical-reactive auxiliaries primarily activate hydroxy groups on the cellulose surface. Usage of reactive chemical auxiliaries notably produces large amounts of hazardous chemicals in the form of halides, hydroxides, and free radicals [14,15,16]. Most commercial methods use alkali-salt solutions [17,18,19,20] for cellulose preactivation. These cellulose preactivation chemicals typically inherit water solubility; therefore, their recycling is not economical at a commercial level in most processes, and these chemicals are disposed of as industrial affluents. One typical example is cellulose-based reactive dyes, which are characteristically known for their high solubility in the water and, therefore, are a significant contributor to textile wastewater, ranked second in the world for water pollution [21].

Reactive dyes can chemically link to hydroxy groups of cellulose in the presence of alkaline. Reactive dyes were developed in the 1950s and have dominated the market since then due to their excellent general fastness properties on cellulose [22,23]. The only shortcoming of cellulose dyeing with reactive dyes is their chemical hydrolysis and degradation in an alkaline solution. The chemical hydrolysis of reactive dyes in water increases rapidly with increasing pH values (i.e., the addition of alkaline salt). Two solutions have been offered to overcome this issue: (a) using bifunctional reactive dyes with two reactive groups to enhance their reactivity with cellulose, and (b) using other green solvents, such as alcohols to reduce the rate of dye hydrolysis [24].

One of the other prominent strategies used to enhance the exhaustion of reactive dyes onto the cellulose materials is the application of 3-chloro-2-hydroxypropyl trimethylammonium chloride (CHPTAC). CHPTAC is commonly used to permanently cationize cellulose to enhance its dyeability. CHPTAC forms an epoxide ring (in situ) in its chemical structure in alkaline conditions (often with the application of NaOH solution), which can be chemically linked to the hydroxy groups of cellulose. The highest fixation rate is achieved at an NaOH:CHPTAC mol ratio above 1.8 [25]. Many textile dyes, including acid, direct, and reactive dyes, are anionic and contain the sodium salt of sulfonate groups. Thus, cationic cellulose (materials) can adsorb anionic dyes, mainly through an electrostatic attraction mechanism from an aqueous solution, even at room temperature [26].

The literature review shows that the activation of cellulose for functionalization is required at high concentrations of NaOH, and the search for more efficient functionalization agents continues. We hypothesize that using a triazine scaffold with symmetric reactivity at three different positions (C-2, C-4, C-6) as an intermediatory scaffold can efficiently activate cellulose for functionalization. These trifunctional reagents can react quickly with the hydroxy groups of cellulose and provide two remaining reactive sites in each molecule for the second step of functionalization with various nucleophile-containing molecules covalently. We predict that a wide range of nucleophile-containing molecules with -NH_2_, -SH, or -OH groups can be used for the second step of cellulose functionalization.

Our preactivators were synthesized from trichlorotriazine, which is widely used in the synthesis of textile reactive dyes. We substituted three chloro groups of trichlorotriazine with three leaving groups using pyridine (PrAct-1), *N*-methylimidazole (PrAct-2), and nicotinic acid (PrAct-3). Structurally, these preactivators contain three cationic centers of triazine (at C-2, C-4, and C-6 positions), demonstrating a distinctive mechanistic chemistry from other commonly used cationic agents for cellulose materials (such as CHPTAC). Those cationic agents increase the cellulose surface charge and enhance ionic–ionic interactions, whereas our developed preactivators chemically link cellulose hydroxy groups with a wide range of amine-containing chemicals.

**Scheme 1:**
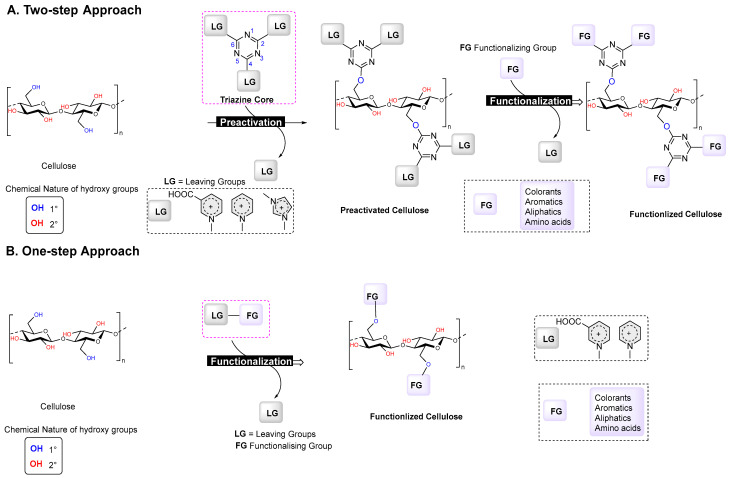
Cellulose functionalizations. (**A**). Two-step approach. (**B**). One-step approach.

To shorten the functionalization procedure of cellulose, we also developed a series of functional materials with the same concept of leaving group chemistry (e.g., Act-1, Act-2, Act-3, and Act-4, as shown in Scheme 1B). These reactive agents can also efficiently functionalize cellulose in one step. The synthesis procedure, purification, and characterization of novel reactive agents are provided in this paper. The functionalized cellulose is characterized by a different method to show the success of our novel two-step and one-step cellulose functionalization (Scheme 1).

The presented paper offers a new and facile strategy for functionalizing cellulose using chemicals containing -OH or -NH_2_ groups, including various types of colorants, peptides, non-aromatic and aromatic hydrocarbons. This distinctive approach can achieve efficient surface functionalization of cellulose via leaving group chemistry while avoiding harmful chemicals. Additionally, this paper provides evidence for the success of this approach using multiple characterization techniques, including zeta potential, reflectance spectroscopy, and time-gated Raman and FTIR spectroscopy. This novel single- and two-step functionalization approach can revolutionize the field of cellulose functionalization and its potential applications.

## 2. Materials and Methods

### 2.1. General Materials and Methods

#### 2.1.1. Chemicals and Instruments

All solvents (acetone, ethanol, methanol, dichloro methane, tetrahydrofuran, acetonitrile, pyridine), reagents (cyanuric chloride, nicotinic acid, *N*-methyl imidazole, *N*-(3-chloro-2-hydroxypropyl)-*N*,*N*,*N*-trimethyl ammonium chloride, 2-hydroxyethyl trimethylammonium chloride, cysteine, Zein protein, 2-aminoanthraquinone, 2-amino-3-methyl-anthraquinone, 1,5-pentandiol, 1-amino-pentane, 1-hexamine, octamine, 1-nanol, benzylamine, benzyl alcohol, phenol) with at least ≥98% purity, and deuterated solvents (CDCl_3_, CD_3_OD, (CD_3_)_2_SO, D_2_O) were purchased from Sigma-Aldrich and Tokyo Chemical Industry (TCI) and used without further purification.

The bleached knitted cotton fabric used in this study was sourced from Orneule Knitting Company in Tampere, Finland. The fabric was a single jersey with a course-24 Ne, wale-24 Ne, and an aerial density of 150 g/m^2^. It was composed of 100% cellulose and did not contain any hemicellulose or lignocellulose components.

#### 2.1.2. Chromatography

Thin layer chromatography (TLC) was used to monitor the synthesis of the chemicals. Merck TLC RP-18 Silica gel 60 F_254_ plates were used. The cylindrical-shaped glass TLC chamber (length and diameter: 8 (5 cm)) was used to run the TLC plates. For the purification of the synthesis of preactivators and activators, RP-18 silica gel (230–400 mesh) was used for gradient column chromatography. A sintered glass disc containing a column with a length and diameter of 80 cm by 3 cm was used to isolate the yields.

#### 2.1.3. NMR Spectroscopy

^1^H, ^13^C NMR spectroscopy, and two-dimensional (COSY, HSQC, and HMBC) experiments were recorded using a Bruker NMR Spectrometer AV III 400 MHz equipped with a 5 mm liquid-state probe. The chemical shifts were recorded in ppm relative to SiMe_4_. The ^1^H and ^13^C NMR data were collected at 400 MHz and 100 MHz for all the compounds, respectively. Deuterated solvents (CDCl_3_, CD_3_OD, (CD_3_)_2_SO, D_2_O) were used for the NMR spectroscopy. The deuterated solvents were used for homonuclear lock, and the signals were referenced to the deuterated solvent peaks. NMR assignments were made by correcting the relative peak of the deuterated solvent. All the processing was performed in MestreNOVA version 11.0 (www.mestrelab.com/, accessed 28 December 2023). The solution was refluxed for 1 h. Acetone was decanted, and the PrAct-1 preactivated fabric was washed with fresh acetone (2 × 5 mL) to remove any surface deposition. The preactivated fabric was then treated with functionalizing agents (choline chloride), and the solution was refluxed for another 1 h in acetone. The functionalized fabric was washed with fresh acetone (2 × 5 mL) to remove any surface solid deposition. Later, the functionalized fabric was treated with ionic liquid for another 24 h to obtain a dissolved cellulose form. After obtaining a dissolved cellulose form, the NMR was recorded, and a postNMR baseline correction was performed.

#### 2.1.4. Zeta Potential Measurements

The zeta potential was measured using a Zetasizer Nano-ZS90 (Malvern). Initially, the sensor tip was washed with deionized water. Later, the zeta potential was measured for a standard (zeta potential transfer standard, −40 mV ± 5.8 mV, size 300 nm) using the dip cell method with a pH of 7.0. After standardizing the instruments, the zeta potential for the samples was recorded. For every sample, three readings were recorded. To prepare the samples for zeta potential measurements, a fabric sample (0.5 g) was pulverized using a milling machine connected to a motor. The particle sized was filtered using 60 Å sieve-sized mess. The collected sample then was subjected to stirring in an appropriate solvent overnight at room temperature.

#### 2.1.5. Reflectance Spectroscopy

The reflectance (R) of the colored samples (i.e., fabric samples functionalized with colorants) at several different points was measured using a GretagMacbeth SpectroScan spectrophotometer. The color strength of the samples (K/S values) was calculated using the *Kubelka–Munk* Equation (1) at a maximum absorption wavelength. The K/S values of the functionalized samples with colorants were obtained before and after washing to estimate the color fixation % using Equation (2). This shows the percentage of the colorants fixed on cellulose by covalent bonds [27].

The coloration properties were measured based on the color strength using the following formula:

(1) $$\mathrm{Color}\;\mathrm{Strength}\;\left(K/S\right)=\frac{{\left(1-\;R\right)}^{2}}{2R}$$

(2) $$\mathrm{Color}\;\mathrm{Fixation}\;\left(\%\right)=\frac{\left(K/S\right)\mathrm{after}\;\mathrm{washing}}{\left(K/S\right)\;\mathrm{before}\;\mathrm{Washing}}\times 100$$

where K represents the absorbance coefficient (which is an intrinsic property), S represents the scattering coefficient, K/S represents the color strength, and R represents the reflectance ratio measured at the maximum absorbance of the colorant.

#### 2.1.6. Fourier-Transform Infrared Spectroscopy

The Fourier-transform infrared (FTIR) spectra were collected using an FT-IR spectrometer (Spectrum Two, PerkinElmer, Shelton, CT, USA) equipped with an ATR unit and a diamond crystal. The spectra were acquired within the wavenumber region of 4000–600 cm^−1^ at a resolution of 4 cm^−1^ and 10 accumulations. The spectra were ATR, and the baseline was corrected using the Spectrum 10 software (PerkinElmer, USA).

#### 2.1.7. Time-Gated Raman Spectroscopy

The Raman spectra were collected using a time-gated Raman spectroscope with a 532 nm frequency-doubled Nd:YAG laser and a DU970-BV EMCCD camera behind a 600 lines/mm grating. The measurements were taken using a 20 × air objective (numerical aperture 0.4; coverslip correction 0.17 mm), an integration time of 0.2 s, and 30 accumulations. The Raman signals outside the 200–3620 cm^−1^ range were excluded. Initially, cyclohexane was used as a standard to evaluate the system’s accuracy. A baseline correction was performed by fitting a fifth-order polynomial over the selected wavenumber range using the WITec Suite 5.1 software. Vector normalization was performed by calculating the sum of the squared intensity values within the selected wavenumber range and using the root of this sum as the normalization factor. The band heights were determined for the bands at ca. 379 and ca. 1091 cm^−1^ to calculate the 379/1091 intensity ratio. This ratio was calculated based on three Raman spectra per sample.

### 2.2. Synthesis, Purification, and Characterization

**Figure 1:**
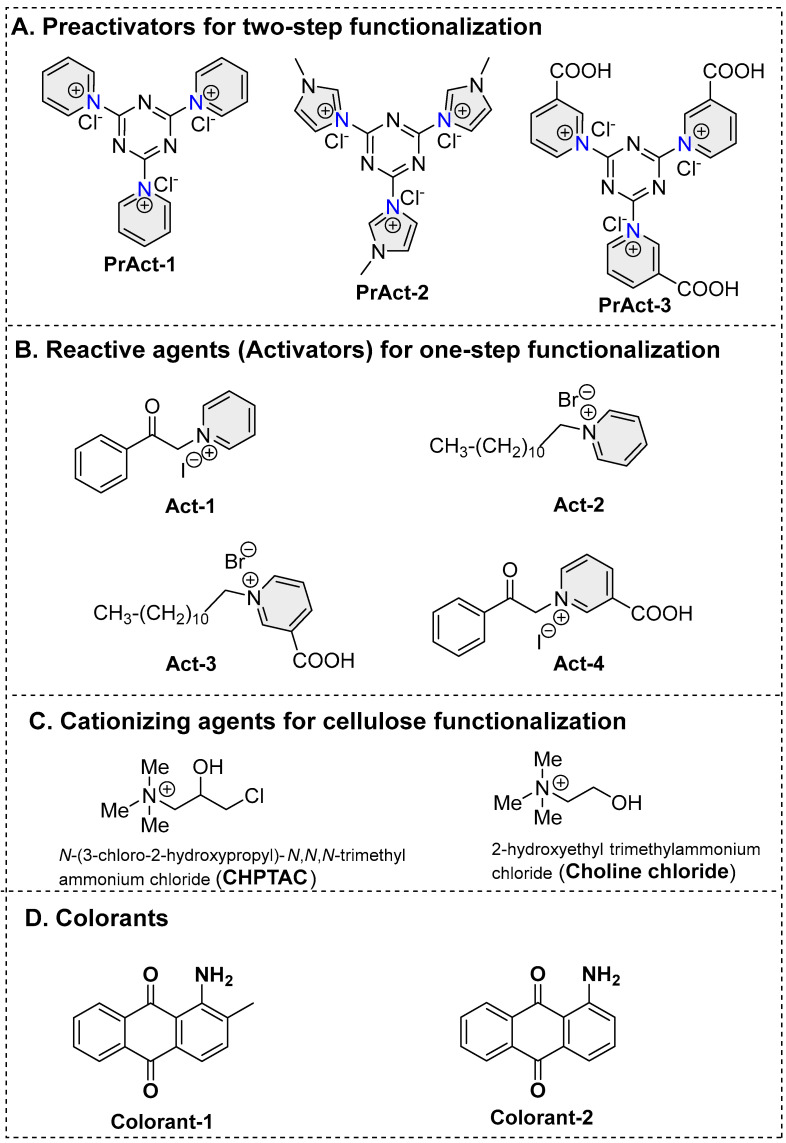
The chemical structures of (**A**) synthesized preactivators (PrAct-1, PrAct-2, and PrAct-3), (**B**) synthesized activators (Act-1, Act-2, Act-3, and Act-4), (**C**) cationizing agents used to study the optimization and strength of preactivators, and (**D**) amine-containing colorants.

The structures of the chemicals used in devising the reaction optimization were as follows: preactivators (two-step activators), one-step activators, cationizing agents, and side products are summarized in Figure 1.

#### 2.2.1. 1,1′,1″-(1,3,5-Triazine-2,4,6-triyl)tris(pyridin-1-ium) (PrAct-1)

To the solution of cyanuric chloride (1 g, 5.42 mM, 1 equivalent) in acetone solvent (20 mL), in a three-necked 25 mL round bottom flask, pyridine (1.31 mL, 16.26 mM, 3 equivalent) was gradually added in a period of 4–5 min due to the exothermic nature of the reaction. After stirring at room temperature overnight, an off-white precipitate was formed, washed with fresh acetone (2 × 10 mL), and filtered under a vacuum (weight = 3.42g, yield = 88%, m.p. = 234–238 °C). ^1^H NMR (400 MHz, DMSO-*d*_6_, ppm) δ 8.91–8.87 (m, 6H, 2-pyridinyl H-2 and H-6; 4-pyridinyl H-2 and H-6; 6-pyridinyl H-2 and H-6), 8.50 (m, 3H, 2-pyridinyl H-4; 4-pyridinyl H-4; 6-pyridinyl H-4), 8.05–7.92 (m, 6H, 2-pyridinyl H-3 and H-5; 4-pyridinyl H-3 and H-5; 6-pyridinyl H-3 and H-5). ^13^C NMR (101 MHz, DMSO-*d*_6_) δ 162.14 (triazine, C-2, C-4 and C-6), 144.89 (-CH-, 2-pyridinyl C-4, 4-pyridinyl C-4, 6-pyridinyl C-4), 142.74 (-CH-, 2-pyridinyl C-2 and C-6, 4-pyridinyl C-2 and C-6, 6-pyridinyl C-2 and C-6), 126.86 (-CH-, 2-pyridinyl C-3 and C-5, 4-pyridinyl C-3 and C-5, 6-pyridinyl C-3 and C-5).

#### 2.2.2. 3,3′,3″-(1,3,5-Triazine-2,4,6-triyl)tris(1-methyl-1H-imidazol-3-ium) (PrAct-2)

To the solution of cyanuric chloride (1 g, 5.42 mM, 1 equivalent) in acetone solvent (20 mL) in a three-necked 25 mL round-bottom flask, *N*-methylimdiazole (1.33 g, 16.26 mM, 3 equivalent) was gradually added over a period of 4–5 min. After stirring at room temperature overnight, a light-yellow precipitate formed, which later was ashed with fresh acetone (2 × 10 mL), and dried (weight = 3.71 g, Yield = 91%, m.p. = 217–221 °C). ^1^H NMR (400 MHz, CD_3_OD, ppm) δ 7.58 (dt, *J* = 13.4, 1.7 Hz, 6H, H-4′ and H-5′ of *N*-methyl imidazole ring; H-4′ and H-2′ of imidazole ring), 3.97 (s, 9H, CH_3_- groups of *N*-methyl imidazole). ^13^C NMR (101 MHz, CD_3_OD, ppm) δ 150.16 (triazine, C-2, C-4 and C-6), 135.59 (-CH-, 2-*N*-methyl-imidazole C-2, 4-*N*-methyl-imidazole C-2, 6-*N*-methyl-imidazole C-2), 123.13 (-CH-, 2-*N*-methyl-imidazole C-5, 4-*N*-methyl-imidazole C-5, 6-*N*-methyl-imidazole C-5), 119.58 (-CH-, 2-*N*-methyl-imidazole C-4, 4-*N*-methyl-imidazole C-4, 6-*N*-methyl-imidazole C-4), 34.81 (-CH_3_-, 2-*N*-methyl-imidazole C-4, 4-*N*-methyl-imidazole C-4, 6-*N*-methyl-imidazole C-4).

#### 2.2.3. 1,1′,1″-(1,3,5-Triazine-2,4,6-triyl)tris(3-carboxypyridin-1-ium) (PrAct-3)

To the solution of cyanuric chloride (0.5 g, 2.71 mM, 1 equivalent) in acetone solvent (20 mL) in a three-necked 25 mL round bottom flask, nicotinic acid (0.996 g, 8.10 mM, 3 equivalent) was added gradually over a period of 2–3 min, and a less exothermic reaction was observed compared to the pyridine and N-methyl imidazole reactions. After stirring at room temperature overnight, a light-yellow compound precipitated, which was washed with fresh acetone (2 × 10 mL) (m.p. = 246–249 °C). ^1^H NMR (400 MHz, CD_3_OD, ppm) δ 9.33 (s, 3H), 9.12–9.05 (m, 3H), 9.03 (q, *J* = 3.6 Hz, 3H), 8.25–8.13 (m, 3H). ^13^C NMR (101 MHz, CD_3_OD, ppm) δ 162.89, 150.16, 146.07, 144.89, 143.24, 130.84, 127.24.

#### 2.2.4. 2-Pyridinium Methyl Phenyl Ketone (Act-1)

To the solution of acetophenone (0.5 mL, 4.28 mM, 1 equivalent) dissolved in acetone solvent (15 mL) in a 25 mL round-bottom flask, nicotinic acid (0.5266 g, 4.28 mM, 1 equivalent) was added and refluxed for 12 h. The acetone was removed under reduced pressure, while reverse-phase column chromatography using a solvent system (H_2_O:MeOH = 9:1) yielded an elute light-yellow compound (yield = 58%, m.p = 203–205 °C). ^1^H NMR (400 MHz, CH_3_OD) δ 9.00–8.90 (m, 1H, phenyl), 8.82–8.70 (m, 1H, phenyl), 8.31–8.20 (m, 1H, phenyl), 8.15 (d, *J* = 7.1 Hz, 1H, phenyl), 7.79 (t, *J* = 7.5 Hz, 1H, phenyl), 7.66 (t, *J* = 7.8 Hz, 2H, phenyl). The ^1^H-NMR data and melting point were in a good agreement with the reported data [28].

#### 2.2.5. 2-Pyridinium Methyl Decanyl Ketone (Act-2)

*Method-1*. To the solution of 1-bromodecane (1 mL, 4.17 mM, 1 equivalent) dissolved in toluene solvent (15 mL) in a 25 mL round-bottom flask, pyridine (0.35 mL, 4.28 mM, 1 equivalent) was added and refluxed for 8 h. The toluene was removed under reduced pressure, and phase column chromatography with a solvent system (H_2_O: MeOH = 5:5) was used to elute the Act-2 as an off-white colored compound (yield = 41%, m.p. = 91–94 °C). ^1^H NMR (400 MHz, DMSO-*d*_6_, ppm) δ 9.19–9.05 (m, 2H), 8.61 (tt, *J* = 7.7, 1.4 Hz, 1H), 8.26–8.01 (m, 2H), 4.61 (t, *J* = 7.5 Hz, 2H), 1.90 (p, *J* = 7.3 Hz, 2H), 1.34–1.15 (m, 18H), 0.88–0.72 (t, 3H).

*Method-2*. The 1-bromodecane (0.5 mL, 4.17 mM, 1 equivalent) was dissolved in pyridine (5 mL) in a 25 mL round-bottom flask and refluxed for 4 h. After cooling the solution in an ice bath for close to 30 min, a yellow precipitate was filtered under a vacuum through a scintillated Buchner funnel, washed with fresh acetone, and dried in the open air (yield = 66%, m.p. = 90–94 °C). ^1^H NMR (400 MHz, CH_3_OD, ppm) δ 9.23–8.89 (m, 2H), 8.61 (td, *J* = 7.7, 3.5 Hz, 1H), 8.14 (q, *J* = 5.8 Hz, 2H), 4.65 (dd, *J* = 12.4, 5.9 Hz, 2H), 2.03 (s, 2H), 1.45–1.33 (m, 4H), 1.30 (d, *J* = 6.4 Hz, 14H), 0.90 (t, *J* = 6.7 Hz, 3H). The ^1^H-NMR data and melting point were in good agreement with the reported data [29].

#### 2.2.6. 3-Carboxy-1-Dodecylpyridin-1-Ium (Act-3)

To the nicotinic acid (0.332 g, 2.70 mM, 1 equivalent), 1-bromodecane (2 mL in the duration of 5 min) was added dropwise, and the reaction mixture was refluxed for 6 h. After cooling the solution in an ice bath for close to 30 min, a pale-white precipitate was filtered under a vacuum through a scintillated Buchner funnel and washed with fresh acetone (2 × 10 mL), with a yield = 73%, m.p. = 186 °C. ^1^H NMR (400 MHz, DMSO-*d*_6_, ppm) δ 9.59 (s, 1H), 9.33 (d, *J* = 6.1 Hz, 1H), 8.95 (dt, *J* = 8.1, 1.5 Hz, 1H), 8.28 (dd, *J* = 8.1, 6.1 Hz, 1H), 4.71 (t, *J* = 7.5 Hz, 2H), 1.92 (m, 2H), 1.29–1.27 (m, 4H), 1.26–1.24 (m, 14H), 0.94–0.77 (m, 3H). The ^1^H-NMR data were in good agreement with the values reported data [30].

#### 2.2.7. 2-Nicotinyl Methyl Phenyl ketone (Act-4)

To acetophenone (0.5 mL, 4.28 mM, 1 equivalent) in a 5 mL round-bottom flask, nicotinic acid (0.474 g, 3.58 mM, 0.9 equivalent) was added and refluxed for 3 h. The reaction was monitored using thin-layer chromatography, and the nicotinic acid was consumed entirely. Reverse-phase column chromatography with a solvent system (Volume ratio = H_2_O: MeOH = 9:1) was used to elute the yellow compound (yield = 68%, m.p. = 235–238 °C) ^1^H NMR (400 MHz, CD_3_OD, ppm) δ 9.09 (dd, *J* = 2.1, 0.9 Hz, 2H), 8.70 (dd, *J* = 5.0, 1.7 Hz, 2H), 8.38 (dt, *J* = 8.0, 1.9 Hz, 2H), 7.53 (ddd, *J* = 8.0, 4.9, 0.9 Hz, 2H). The ^1^H-NMR data and melting point were in good agreement with the reported values reported data [31].

### 2.3. Two-Step Functionalization of Cellulose

#### 2.3.1. Cellulose Preactivation (Step 1)

The cotton fabrics (1 g) were soaked in different percentages of solution (0.5%, 1%, 2%, 5%, 10%) with preactivators (PrAct-1, PrAct-2, and PrAct-3) in acetone (25 mL) and at different temperatures for 30 min. This preactivated cotton fabric was washed with fresh solvent (2 × 10 mL), and oven-dried at 60 °C for 1 h.

#### 2.3.2. Functionalization of the Preactivated Cellulose Material (Step 2)

The preactivated cellulosic samples were treated with nucleophile-containing chemicals, including colorants, aromatic and long-chain hydrocarbons, amino acids (cysteine), and biopolymers (zein protein) under various conditions.

##### Functionalization with Choline

The preactivated cellulosic material (cotton fabric, 100 mg) with the preactivators was treated with choline chloride at different concentrations (0.5, 1, 2.5, 5, 10 *w*/*w*%) using 25 mL of various solvents (acetone, methanol, ethanol, ethyl acetate, isopropyl alcohol, propanol, dioxane) and a temperature of 60 °C for 30 min.

##### Functionalization with Colorants

The preactivated cotton fabrics were colored with two colorants. These amine-containing anthraquinone colorants (colorant-1: 1-amino anthraquinone, C_15_H_11_NO_2_, molecular weight = 237.08; colorant-2: 1-amino-2-methyl anthraquinone, C_14_H_9_NO_2_, molecular weight = 223.06) were ideal for this study. The coloration was achieved using a dyeing machine, where the experiment was divided into parts. In part-a, preactivation was performed using acetone (25 mL), T_activation_ = 50 °C for 30 min, while in part-b, 5% *w*/*w* of colorants (1 or 2) was added to the dyeing bath, and dyeing was performed at 50 °C for 30 min. The fabrics were then washed with fresh acetone (3 × 25 mL). Later, these samples were processed for their coloration characteristics and measured using reflectance spectroscopy. The reflectance spectroscopy helps in estimating the fixation of coloration (Equation (2)) onto the cotton fabrics before and after dyeing and therefore serves as an efficient way of evaluating the efficacy of the preactivation compounds compared to the samples for which no preactivation compounds were used.

##### Functionalization with Amino Acids and Peptides

The preactivated cotton fabrics (100 mg) were treated with cysteine (5% *w*/*w*) in a reaction flask containing acetone as a solvent (25 mL), which was refluxed for 30 min. After 30 min, the reaction vessel was cooled down, the acetone was decanted, and the treated cotton fabrics were washed with fresh acetone (2 × 10 mL). For the case of zein protein, initially, a solution of 5% was prepared in a 25 mL 1:1 mixture of acetone:ethanol (which had 0.01 sodium carbonate) to activate the zein amino groups. To this solution, the cotton fabric activated with PrAct-1 (100 mg) was soaked for 5–6 min and then refluxed for 30 min. After refluxing, the solvent was decanted, and fresh acetone (3 × 10 mL) was used to wash the zein-functionalized cotton fabric.

##### Functionalization with Aromatic and Aliphatic Moieties

To the soaked cellulose fiber or cotton fabrics (100 mg) in 25 mL of acetone, 5% preactivation compounds (either PrAct-1 or PrAct-2 or PrAct-3) was added and heated at 60 °C for 30 min. After that, 10% (*w*/*w* of cotton fabric) aromatic and aliphatic moieties were added under reaction conditions of 60 °C for 30 min. Later, the reaction liquid (solvent) was initially decanted, and the remnants were removed under reduced pressure (using a mouth gauge). To remove the excess of aromatics or non-aromatics, which are deposited on the surface, the cellulose fibers were washed with 3 × 10 mL of fresh acetone. Later, these functionalized fibers were characterized using Raman spectroscopy, FTIR, zeta potential, and solubilized NMR.

### 2.4. One-Step Functionalization of Cellulose

To the solution of cotton fabric (100 mg) in 25 mL of acetone, 5% (*w*/*w* of cotton fabric) activator concentration (either Act-1 or Act-2 or Act-3 or Act-4) was refluxed for 30 min. The fabric sample was removed from the reaction and washed with fresh acetone (2 × 10 mL). Later, the cotton fabrics were washed with non-ionic soap, air-dried, and characterized for specific fingerprint region peaks using FT-IR spectroscopy.

## 3. Results

### 3.1. Chemistry

**Scheme 2:**
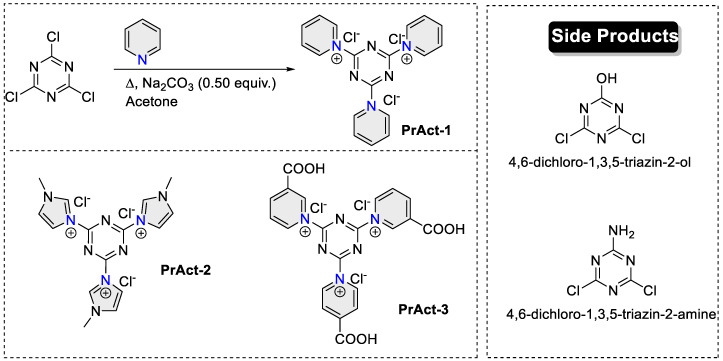
Synthetic route devised for the development of preactivators and side products.

Attaining three nucleophilic additions onto an aromatic ring to form a quaternary salt using *N*-heterocycles is challenging; therefore, electronegativity of such aromatic systems as starting materials plays a critical role. Thus, highly electronegative substituents containing aromatic systems are commonly used; for example, triazine substituted with halides demonstrates sufficient electronegativity for facilitating the nucleophilic substitution at its C-2, C-4, and C-6 positions [32,33]. As these reactions are prime examples of the S_N_Ar type, the choice of solvents and localized lone pairs of the aromatic nitrogen atom of *N*-heterocycles also play an essential role in deciding the kinetics of the reaction [34]. However, nucleophilic substitution reactions onto cyanuric halides are moisture-sensitive and are commonly known to increase the overall halide load of the reaction. For instance, in our observations, the reaction between cyanuric chloride and pyridine in MeOH leads to the formation of a side product (4,6-dichloro-1,3,5-triazin-2-ol: HRMS-ESI: *m*/*z* calc for C_3_H_2_Cl_2_N_3_O: 165.9569, found: 165.9576 [M + H]^+^ (within 4.21 ppm)). Therefore, we have not evaluated the protic solvents during reaction optimization (including MeOH, EtOH, BuOH, i-*Pr*-OH, and 1-Pr-OH). Our choice of *N*-heterocycles and choosing pyridine as the leaving group is based on the fact that it is an easily accessible solvent and is economically cheaper than other *N*-heterocyclics. Interestingly, Cheng et al. have already investigated exploiting pyridine as a leaving group [35]. In their study, they found a mixture of two regioisomers (benzopyridothiazepine and benzo[*b*]thiophenes). Such products were believed to be a result of two competing cyclization reaction pathways ([5 + 2] and [3 + 2]), where pyridinium 1,4-zwitterionic thiolates (as precursors) were cyclized with arynes. Interestingly, pyridine was proposed as the leaving group in the [3 + 2] reaction pathway to form benzo[*b*]thiophenes but not in the [5 + 2] pathway, where pyridine itself becomes fused with a seven-membered ring to form benzopyrido [1,2-*d*][1,4]thiazepines [35]. Notably, the [3 + 2] cascade cyclization reaction occurs via a *S*-nucleophilic addition, *C*-Michael addition, and *retro*-Michael addition, where pyridine behaves as a leaving group [35]. Their study also revealed solvent utilization, where aprotic polar solvents (THF, acetonitrile, DCM) showed superior conversions, while nonpolar solvents (toluene) had a low conversion rate. Similar observations were also found in our experiments, where aprotic polar solvents demonstrated a higher conversion than nonpolar solvents. In our opinion, the solubility of cyanuric chloride and pyridine in aprotic protic solvents against nonpolar solvents is, to an extent, responsible for such observations. Secondly, an additive in the form of a mild base was introduced to mitigate the halide load onto the reaction. It has been previously observed that halides (as leaving groups in the form of side products) compete for nucleophilic reactions with upcoming nucleophiles depending on the strength of the localized electrons on the heteroatom of the nucleophile and the molecular obesity that also restricts the attack angle due to higher steric hindrance. A clear difference between the yields resulted from potassium carbonate (K_2_CO_3_) facilitated reaction versus those where it was not used, demonstrating the importance of K_2_CO_3_ as additive for these reactions. However, using ammonia methanol as an additive mixture leads to the formation of 4,6-dichloro-1,3,5-triazin-2-amine, showing the cross-reactivity of cyanuric chloride for amine-containing bases, as shown in Scheme 2.

**Scheme 3:**
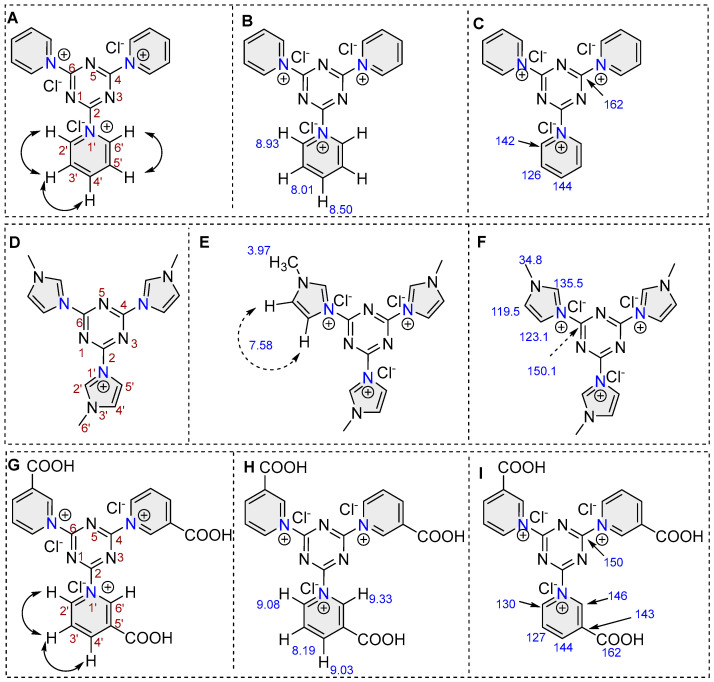
Structural features indicating the numbering of atoms for ^3^J_HH_ COSY, ^1^H-NMR, and ^13^C-NMR, and the correlation of vicinal protons in PrAct-1 (**A**–**C**), PrAct-2 (**D**–**F**), and PrAct-3 (**G**–**I**).

However, an increase in temperature explicitly increases the trimeric substation of cyanuric chloride. Based on the evaluation, the reaction conditions were optimized (as shown in Table 1 and Scheme 2), and as a result, three preactivators were synthesized (PrAct-1, PrAct-2, and PrAct-3). To confirm the synthesis of compoundshe ^1^H-NMR, ^13^C-NMR, COSY (vicinal protons (^3^J_HH_) were correlated) spectra were used to confirm the synthesis of these preactivators (Scheme 3).

### 3.2. Cellulose Treatment with Preactivators

**Figure 2:**
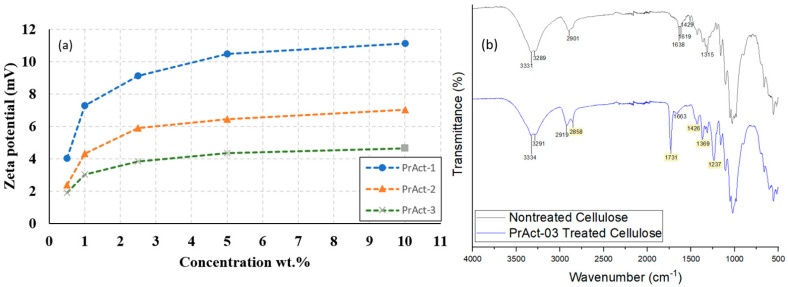
(**a**). Chemical reactivity: compared zeta potentials of preactivators with cellulose, including the zeta potential of the cellulose samples after reaction with preactivators at various concentrations. The zeta potential of the untreated cellulose sample was −9.63 mV. (**b**). A comparison of FTIR dips of nontreated cellulose with PrAct-3-treated cellulose (showing COOH stretching, C=C aromatic stretching, C-H stretching, and O-H stretching).

Various concentrations of PrAct-1, PrAct-2, and PrAct-03 were used to preactivate the cellulose (% weight over the weight of the cotton fabric = 0.5, 1, 2, 5, and 10). The measured ζ-potential for each sample was plotted using a 2D scattering plot as shown in Figure 2a. The untreated cellulose sample had a ζ-potential of −9.63 mV, which gets neutralized with all the preactivators at a 0.5% *w*/*w* of the cotton fabric. This shows that the preactivators can cationize cellulose very efficiently, even at low concentrations. We noticed that PrAct-1 cationizes cellulose much more efficiently and at much lower concentrations than the other two preactivators. The substantial difference in the cellulose surface cationization of PrAct-1 over PrAct-3 can be understood based on their chemical reactivities, which reflects a significant difference in their physicochemical properties. The presence of electronegative COOH groups in the chemical structure of PrAct-3 might affect the solubility and have a steric repulsion from the lone pairs of the (oxygen atoms) polyhydroxy groups of cellulose. At a 10% *w*/*w* concentration, the PrAct-1 showed a ζ-potential of +11.12 mV, followed by PrAct-2 (ζ-potential = +7.02 mV) and PrAct-3 (ζ-potential = +4.65 mV). Comparing the ζ-potential among these preactivators (at 10% *w*/*w*) demonstrated their ability to cationize cellulose, which also reflects their cellulose chemical reactivities.

Based on Figure 2a, an overall comparison among the preactivators for cellulose chemical reactivity can be distinguished in the following order: PrAct-01 > PrAct-2 > PrAct-3. For comparison, we also treated cellulose with a commercially available cationizing agent (i.e., CHPTAC) at 10 wt.%. The zeta potential of the sample was found to be +4.08 mV, which could be achieved by all the preactivators at much lower concentrations. This can be explained by: (a) steric hindrance of aromatic rings as leaving groups versus the smaller aliphatic nature of CHPTAC, (b) the dicationic nature of the PrAct series versus the monocationic nature of CHPTAC, and (c) the requirement of NaOH activation for the preactivation reaction of CHPTAC with cellulose, where NaOH itself can reduce the quaternized amino groups of CHPTACs. However, the FTIR spectra of all these preactivators showed subtle differences, while more evident peak separation was clearly observed for the COOH group (sharp, C=O stretching at 1731 cm^−1^) of Pract-3 (as shown in Figure 2b). Other distinctive peak differences are visualized, such as the C-H stretching at 2880–2950 cm^−1^ and broad O-H stretching, as shown in Figure 2b.

### 3.3. Optimization of Preactivation-Treated Cellulose with Functionalizing Agents

Initially, we attempted to functionalize the PrAct-03 preactivated cellulose fibers with choline chloride. Choosing choline chloride for preactivated cellulose was based on the abundant presence of aliphatic methyl (-CH_3_) groups in the choline chloride structure, whose peaks do not overlap with the proton peaks of glucose units (of cellulose) and usually have a sharp intensity in ^1^H-NMR spectroscopy. However, no clear inferences were made when choline chloride-functionalized cellulose fibers were studied using solution NMR (treated cellulose fibers dissolved using ionic liquid Tetrabutylphosphonium acetate P4444(OAc)). One of the primary reasons for such inferences was the low stoichiometry ratio of the choline chloride that functionalized the preactivated cellulose fibers (even at a high concentration of 10% choline chloride). Therefore, the explicit peaks of the functionalized choline chloride onto the preactivated cellulose fibers were not sharp enough to differentiate from artifact peaks after post-processing and baseline correction of the ^1^H-NMR spectra. Also, missing peak(s) distinctive to the choline-functionalized preactivated cellulose fibers in the Raman spectroscopy, overlapping of C-H stretching of functionalized choline chloride (with cellulose C-H stretching), and N-H stretching of choline chloride (with O-H stretching) in the FTIR spectroscopy rendered them unable to provide any structural information related to the extent of functionalization achieved in these experiments. Therefore, we focused on the cationic nature of choline chloride, which, when functionalized to preactivated cellulose fibers, must change their surface charge (in the form of the zeta potential).

Table 2 shows the zeta potential of the cellulose samples preactivated with PrAct-1,2, and 3 and functionalized with choline chloride (10 wt.%). Choline chloride cannot react with the hydroxy groups of cellulose, and the zeta potential of the samples does not change noticeably. However, the zeta potential of the samples preactivated and treated with choline chloride increased noticeably, demonstrating the success of the two-step functionalization process proposed in this study (Scheme 1A). We used different concentrations of functionalizing agents (0.5%, 1%, 2%, 5%, and 10%). The zeta potential of the preactivated cellulose with 10 wt.% PrAct-1 and after being treated with 10 wt.% choline chloride in various solvents was in the range of 11.55–15.53 mV, and the best result was obtained when acetone was used as the solvent. Compared to PrAct 2 and 3, PrAct-1 was found to be a more efficient preactivator for cellulose functionalization with choline chloride. We have already discussed in the previous section.

The reactivity of choline chloride with preactivated cellulose can further be enhanced by increasing the temperature (Table 3). This enhancement was much more noticeable for the cellulose sample preactivated using PrAct-1. The zeta potential of the samples after treatment with choline chloride was further increased from 15 to 18 mV by increasing the temperature from 25 to 50 °C, respectively. The reaction between choline chloride and preactivated cellulose depends on the rate constants for hydrolysis of the functional groups and the fixation of choline chloride on cellulose, which are both temperature-dependent.

### 3.4. Functionalization of Preactivated Cellulose with Amine-Containing Colorants

As discussed in the previous sections, the extent of functionalization majorly depends on the nucleophilicity of the functionalizing agents; therefore, we selected two amino-containing anthraquinone-based colorants (Figure 1). These two colorants are not water soluble, and they do not have substantivity toward untreated cellulose in water. The untreated and preactivated cellulose fabrics were dyed with colorants 1 and 2 in acetone. The samples were then washed to remove any surface-deposited preactivators and colorants. The fixation % and coloration strength (K/S) of the samples are reported in Table 4. As can be seen, the colorants did not adsorb on the untreated cellulose, and the samples had a pale red color. However, the colorants reacted with the functional groups of the preactivated cellulose, and the samples showed a noticeable color strength. The fixation % of the dyed samples with colorant-1 and colorant-2 were found to be 63.8, and 87.9%, respectively.

Similar profiles were observed when using the colorants (colorant-1 and colorant-2) for nonactivated cellulose, which demonstrated the significance of the preactivation of PrAct-1 of cellulose. The fixation % of colorant-1 and colorant-2 on the preactivated cellulose fabric was found to be 63.8% and 87.9%, respectively. The lower fixation % of colorant-1 can be attributed to the presence of a methyl group in close vicinity of the amine group. This methyl group reduces the reactivity of colorant-1 to functional groups of PrAct-1 due to spatial hindrance. Such steric hindrances by alkyl substituents, where the presence of alkyl group reduces the kinetics of incoming nucleophiles, have been reported earlier [36,37]. The plotted reflectance spectroscopy graphs for colorant-1 and colorant-2 are shown in Figures S11 and S12.

### 3.5. Functionalization of Preactivated Cellulose with Amino Acids and Peptides

Amino acid-functionalized cellulose platforms have a wide variety of biomaterial applications, such as bioinspired membranes [38], biofilms [39], and biocomposites [40]. In our study, we emphasize using cysteine because of its suitability for our experiments, as it displays the highest nucleophilicity compared to other amino acids [41]. Cysteine is a sulfur-containing amino acid that rapidly dimerizes to form disulfide bonds. As the most nucleophilic amino acid (nucleophilicity measured for dianionic cysteine = 23.43, which is higher than all the other amino acids), it is an ideal choice for nucleophilic addition and substitution reactions. In the FTIR, the cysteine-functionalized cellulose exhibited typical peaks, such as a significant change in stretching vibrations for C-H (2915 cm^−1^), broad -OH stretching (3457 cm^−1^), and C=O of the carboxylic group (1628 cm^−1^) [42]. Interestingly, the visualization of the peak at 1703 cm^−1^ shows cystine’s successful incorporation over the cellulose surface, as shown in Figure S13.

**Figure 3:**
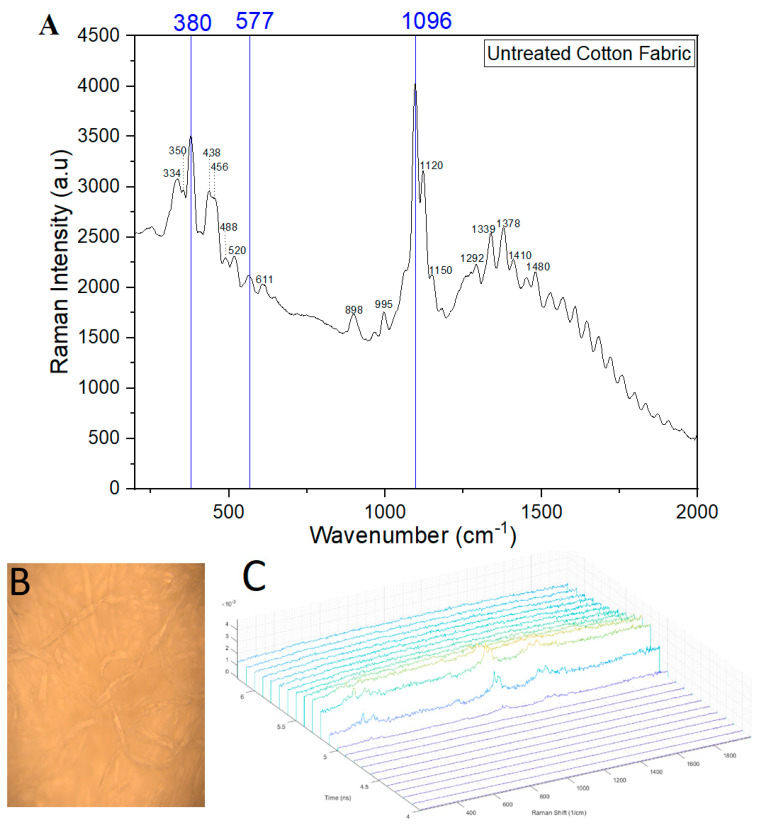

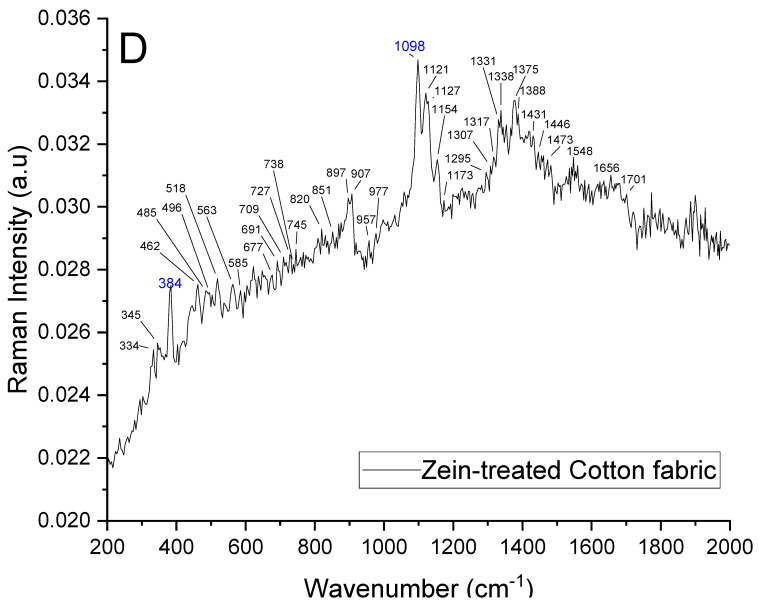
Raman spectral characterization of (**A**) cotton fabric; (**B**) localized fiber (in focus) irradiated with a 532 nm laser; (**C**) time-gated 3D spectra of zein-treated cotton fabric; and (**D**) Raman spectral characterization of zein-treated cotton fabric extracted in the 5.1–5.3 ns range.

The Raman spectra of the cotton fabrics displayed intense bands as stretching vibrations of the C-O-C glycosidic ring breathing at 1096 cm^−1^ (asymmetric). The other specific peaks for cellulose-rich cotton usually appear at 380 cm^−1^ (C-C-C, C-O, and C-C-O ring deformation bending; can be correlated to cellulose crystallinity). A peak at 577 cm^−1^ has a distinctive significance identifying the cellulose-II allomorph compared to cellulose-I (1096 cm^−1^, a distinctive peak representing cellulose-I) [43]. Cotton fabric possesses a relatively high percentage of cellulose-I, which is characterized by the following Raman (bands) peaks: 1096 cm^−1^ (sharp), 1121 cm^−1^ (sharp, representing the stretching, C-C and C-O; ν(COC) in plane symmetric), along with some weaker peak (998 cm^−1^ (Stretching, C-C and C-O; ρ(CH_2_)). In our Raman spectra, we observed the following peaks for nontreated cellulose-rich cotton, which were characterized based on the previous reported literature [43,44,45]), which is also shown in Figure 3A: 334 cm^−1^ (shoulder peak, Heavy atom bending; ring twisting); 350 cm^−1^ (wide peak, some heavy atom stretching); 380 cm^−1^ (moderate peak, some heavy atom stretching; *δ*(C-C-C) ring); 438 cm^−1^ (moderate peak, some heavy atom stretching; *ν*(C-C-O) ring); 456 cm^−1^ (moderate peak) some heavy atom stretching; *ν*(C-C-O) ring; 488 cm^−1^ (weak peak) some heavy atom stretching; *ν*(C-C-O) glycosidic; 520 cm^−1^ (moderate peak) some heavy atom stretching; *ν*(C-C-O) ring glycosidic; 610 cm^−1^ (very weak) *δ*(C-C-H) twisting; 898 cm^−1^ (moderate peak) *ν*(COC) in plane symmetric; 995 cm^−1^ (weak peak) Stretching, C-C and C-O; *ρ*(CH_2_); 1096 cm^−1^ (sharp peak), Stretching, C-C and C-O; *ν*(C-O-C) glycosidic asymmetric; 1120 cm^−1^ (sharp peak) Stretching, C-C and C-O; *ν*(C-O-C) in plane symmetric; 1150 cm^−1^ (moderate peak) heavy atom stretching plus, H-C-C and H-C-O bending; *ν*(C-C) ring breathing asymmetric; 1292 cm^−1^ (moderate peak) H-C-C and H-C-O bending; *δ*(CH_2_) twisting;1339 cm^−1^ (moderate peak) H-C-C, H-C-O, and H-O-C bending; *δ*(CH_2_); 1378 cm^−1^ (moderate peak) H-C-C, H-C-O, and H-O-C bending; *δ*(CH_2_); 1410 cm^−1^ (shoulder peak) H-C-C, H-C-O, and H-O-C bending; *δ*(CH_2_); 1480 cm^−1^ (moderate peak) H-C-H and H-O-C bending; *δ*(CH_2_) scissors.

Zein protein is an alcohol-soluble prolamine protein, found in the range of 2.5–10% (dry weight) in corn. It is commonly employed in the production of coating, adhesives, laminated boards, and color printing films. The solubility of zein limited to alcoholic solvents and usually requires an alkaline pH, which led us to use 0.01N Na_2_CO_3_ during our experiments. The compositions of zein protein depends on the chain (chain-A: glutamine (36.1%), leucine (35%), alanine (21.8%), proline (16.9%) phenylalanine (14.4%), tyrosine (11.8%), valine (11%), while other amino acids are in range of 2.5–9.9%) [46]. Interestingly, methionine and cysteine are the least abundant amino acids in zein protein; therefore S-S, C-S, and S-H stretching are usually less abundant [46].

In our experiment, zein protein-functionalized cotton fabric was compared with nontreated cotton fabric, as shown in Figure 3. The comparative differences showed specific peaks (1701/1656 cm^−1^, 1295 cm^−1^) representing the C=O and amide backbone, confirming zein functionalization onto the cellulose [47]. The appearance of the 1701/1656 cm^−1^ (vibrational mode of amide I) and amide III peaks greater than 1295 cm^−1^ shows that the zein protein primarily adopts the α-helix secondary structure. These peak assignments agree with previous literature reporting that the zein protein molecular structure was found to have a high α-helix content [48,49,50]. In contrast, the presence of aromatic amino acids is quite evident, with peaks at 677 cm^−1^ and 977 cm^−1^, attributed to the presence of phenylalanine. A peak at 977 cm^−1^ represents phenyl ring breathing, which is not conformation-sensitive and can be used as an internal intensity standard. In contrast, the appearance of the 820 and 851 cm^−1^ peaks represent vibrational modes of the phenolic side chain of tyrosine. Such observations indicate the abundance of aromatic amino acids in zein-treated cotton fabric. Furthermore, the significant presence of aromatic amino acids peaks demonstrates that the α-helix in the zein protein is reasonably rigid and stable [50]. Interestingly, the intensity ratio (I_851/820_) of these two peaks helps us to identify the nature of the H-bonding of the phenol hydroxy group [51]. If the tyrosine residue is solvent-exposed in an aqueous alcoholic solution, (a) the phenolic-OH group acts as an acceptor–donor with a weak–moderate H-bond, and the intensity ratio (I_851/820_) will be nearly 1:0.8 (I = 1.25); and (b) the phenolic-OH group acts as an acceptor with a strong H-bond, and the intensity ratio will be 1:0.4 (I_851/820_ = 2.5); (c) the phenolic-OH is the H^+^ donor in a strong H-bond, and the intensity ratio will be 1:2 (I_851/820_ = 0.5). As a result of our characterization, we observed moderate-to-weak peaks at 851 cm^−1^ and 820 cm^−1^. Based on the absolute peak integration, we found an intensity ratio (I_851/820_) = 0.71, suggesting that functionalized zein phenolic groups of tyrosine are modified onto the cellulose surface as the H-donor rather H-acceptor. The other peaks below 600 cm^−1^ represent the pyranose ring bending modes of the cellulose in the cotton fabric. The splitting of 898 cm^−1^ (broad peak, representing the *ν*(COC) in plane symmetric of cellulose) into 897 and 907 cm^−1^ indicated the structural changes after functionalization. The region from 800 to 1700 cm^−1^ of the zein-treated cotton is dominated by asymmetric stretching vibrations of C-O-C glycosidic linkage and H-C-C/H-C-O bending modes of pyranose ring of cellulose. The peak at 957 cm^−1^ is assigned to H-C-C and HCO bending. The zein-treated cotton fabrics showed significant changes in the peak’s shape in the 700–900 cm^−1^ compared to the untreated cellulose-rich cotton peaks, represent the distinctive chemical nature of side chains of various amino acids of zein protein. The peak includes glycosidic bond symmetric stretching, C-C stretching, and C-O stretching of pyranose rings, which shifted after functionalization with the zein protein. The deconvoluted Raman spectra of these regions are shown in Figure 3. The deconvolution suggests the shifting of individual peaks by several wavenumbers and changes in their intensities. The changes in the shape and intensity of the peaks indicate possible substitution of primary hydroxy groups. Similarly, significant differences were also observed in the bending regions of H-C-H, H-C-C, H-O-C, and C-O-H, and the deconvoluted peaks of the area centered at 1295 cm^−1^ (regions of 1150–1600 cm^−1^) were characterized by peaks at 1154, 1338, 1375, 1431, 1446, 1473, 1548 cm^−1^. Furthermore, distinctive differences were recorded in the FTIR, where the zein-treated cotton fabric samples showed changes and the appearance of peaks in the C-H stretching region (2918, 2900, 2849 cm^−1^) and -CO-NH- backbone region (1650–1450 cm^−1^). This indicated the presence of alkyl side chains of non-ionic amino acids and their peptide backbone of the functionalized zein (Figure S14).

### 3.6. Functionalization of Preactivated Cellulose with Hydrocarbons

Cellulose functionalization with hydrocarbons is primarily achieved using silane chemistry, where aminoalkyl silanes are commonly employed [52,53,54,55,56]. However, being an example of ideal cross-linkers, aminoalkyl silanes have inherited vulnerability to yield side products and commonly chelate with the monovalent and divalent metal ions in aqueous solutions; therefore, there is an immense interest among material chemists to develop silane-free chemical methods for cellulose materials.

**Figure 4:**
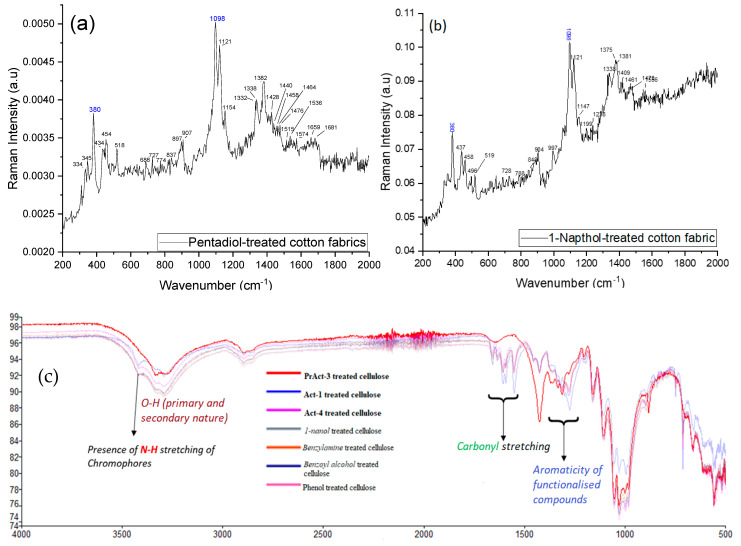
(**a**) Time-gated Raman spectra of 1,5-pentadiol-treated cotton fabric, (**b**) time-gated Raman spectra of 1-Napthol-treated cotton fabric, (**c**) comparative FTIR spectra of PrAct-3 preactivated cellulose fabric, Act-1, and Act-4-activated cellulose fabric with aromatic hydrocarbon-functionalized cellulose fabric.

We treated preactivated cellulose fabrics with the various hydroxy or amine-containing chemicals listed in Table 5. An enhancement in C-H stretching (in the range of 2833–2978 cm^−1^) was a critical FTIR peak commonly found in all the samples, demonstrating the increase in surface alkylation of cellulose. However, subtle changes were observed on the longer side of FTIR (in the range of 3327–3512 cm^−1^), depicting the functionalizing of the hydroxy groups of cellulose. Time-gated Raman spectroscopy of one of the non-aromatic hydrocarbon representative of 1,5-pentadiol-treated cotton fabric showed a CH_2_ bending band and Fermi resonance band at 1338 cm^−1^ and 1382 cm^−1^, as shown in Figure 4a. The spectra region from 900 to 1700 cm^−1^ of the 1,5-pentadiol-treated cotton fabric exhibited peaks characterized by asymmetric stretching vibrations of C-O-C glycosidic linkages and H-C-C/H-C-O bending modes of the pyranose ring [57].

Aromatic compounds, such as benzylamine (which bonds covalently to cellulose through its amino group, C_6_H_5_-CH_2_-*N*H_2_), benzyl alcohol (which bonds covalently to cellulose through its hydroxy group, C_6_H_5_-CO-*O*H), phenol (which bonds covalently to cellulose through its hydroxy group, C_6_H_5_-*O*H tethering), 1-naphthol (which bonds covalently to cellulose through its hydroxy group, C_6_H_4_-C_6_H_3_-*O*H), were treated with preactivated cotton fabrics. Spectroscopy acquisitions were carried out on washed cotton fabric (which was initially washed with acetone and followed with water). In the FT-IR spectroscopy, the prominent aromatic region was observed to preactivate the cellulose in the fabric compared to PrAct-1, as shown in Figure 4c. The time-gated Raman spectroscopy of the first aromatic region representative of 1-naphthol-treated cotton fabric was characterized by a specific peak appearing at 728 cm^−1^, which represents the 1-naphthyl linkage to pyranose. This differentiates the 1-naphthyl substitution from the 2-naphthyl substitution, as shown in Figure 4b [58]. The weak peak at 1556 cm^−1^ and moderate peak at 1462 cm^−1^ represent the C-C stretching and C-H in-plane bending modes, respectively [59]. The peak at 1238 cm^−1^ represents the ring C-C and C-O stretching modes. The relative intensity of the peaks at 1147 and 1199 cm^−1^ represent the different C-C stretching mode and in-plane bending of C-H in the functionalized cellulose form. The observed peaks at 967/904 cm^−1^ represent different C-C out-of-plane bending modes, and a weak peak at 848 cm^−1^ represents the out-of-plane deformation C-H of the functionalized cellulose [60]. A shoulder peak at 788 cm^−1^ represents the O-H out-of-plane deformations, whereas the 728 cm^−1^ peak was assigned to the C-C-C in-plane bending mode. Other peaks for the C-C-C in-plane deformation appeared at 670, 571, and 485 cm^−1^ [59]. However, the stretching vibration bands at 1098 cm^−1^ represent the C-O-C glycosidic ring breathing, while the Raman bands at 380 cm^−1^ (C-C-C, C-O, and C-C-O ring deformation bending) were found to be specific to the cellulose-I form [43]. Other peaks for the C-C-C in-plane deformation appeared at 519, 496, and 437 cm^−1^.

### 3.7. One-Step Cellulose Functionalization

**Scheme 4:**
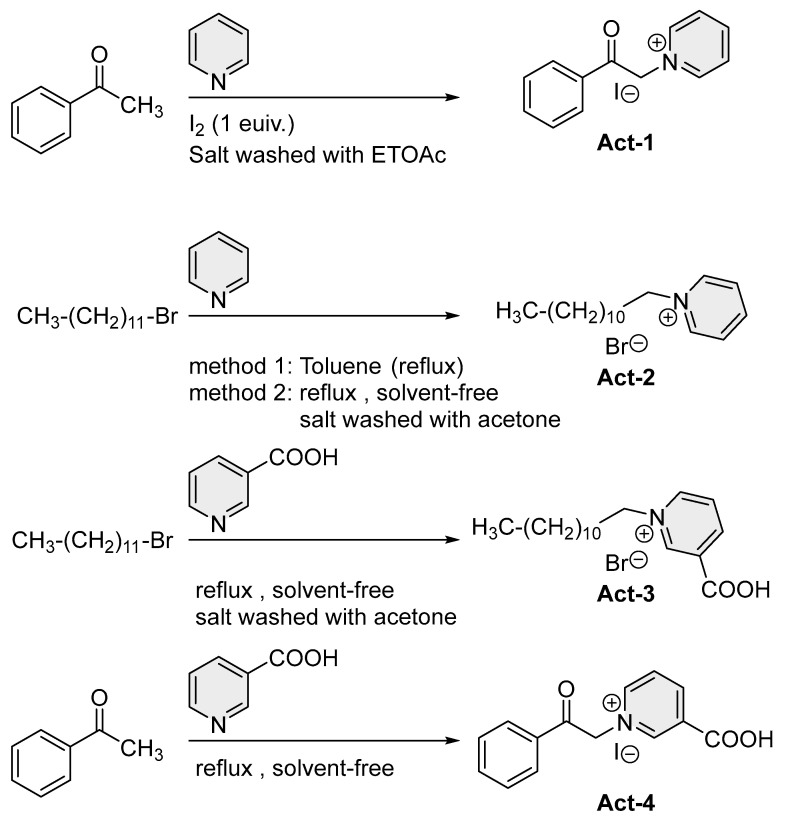
Synthesis routes of four reactive chemicals (Act-1, Act-2, Act-3, and Act-4).

To achieve direct functionalization, we synthesized Act-1, Act-2, and Act-3 by reacting long-chain hydrocarbons and aromatic groups directly with the leaving group (pyridine and nicotinic acid). Activators were developed based on the synthetic route developed for α-pyridinium methyl ketone salts [61] specialized for the triphenyl pyridine core, as shown in Scheme 4.

**Figure 5:**
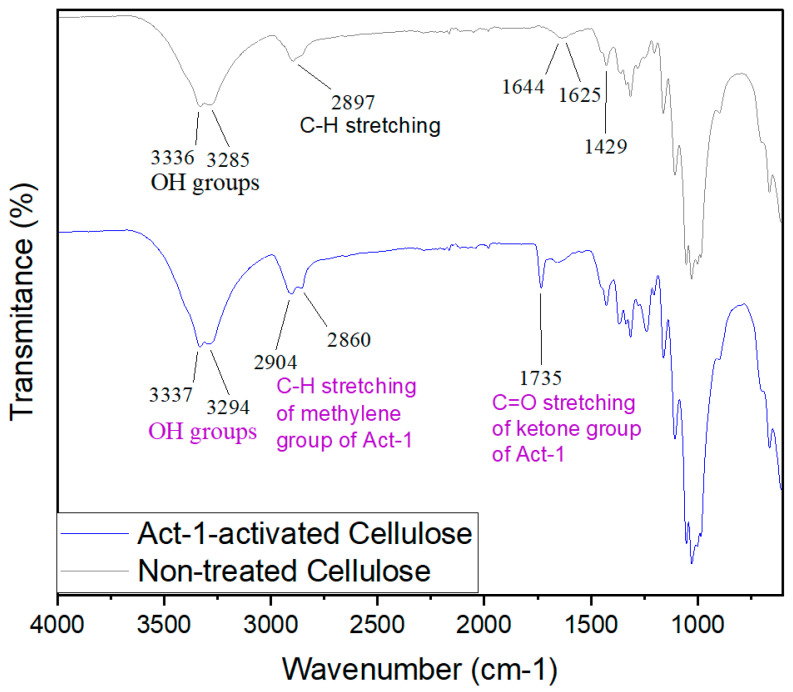
FTIR spectra of nonactivated cellulose fiber (right-hand side) and Act-1-treated cellulose fiber (left-hand side).

The functionalization of cellulose with these activators (Act-1, Act-2, Act-3, and Act-4) was validated using FTIR and time-gated Raman spectroscopy. Act-1-treated cotton fabric showed more significant C-H stretching at 2904 cm^−1^ and aromatic C-H stretching at 1200–1450 cm^−1^ at 50 °C than at room temperature, as shown in Figure 5. More pronounced C-H stretching at 2917 cm^−1^ was observed in the untreated cotton fabric compared to the Act-2-treated fabric, even at room temperature. However, no changes were identified in the C-H stretching and aromatic regions (C-H stretching) when treated with Act-3, even at 50 °C. Interesting, subtle changes were identified in the aromatic region (aromatic C-H stretching at 1200–1450 cm^−1^) when treated with Act-4 at 50 °C compared to the one treated at room temperature.

In our observation, activators (Act-1 and Act-2) containing pyridine as the leaving group attained relatively higher functionalization of cellulose than Act-3 and Act-4-containing nicotinic acid. These results explicitly demonstrate the impact of the nature of the leaving group on cellulose functionalization.

## 4. Conclusions

We have successfully implemented leaving group chemistry to develop strategies that enhance the reactivity of cellulose towards functionalization. Our two-step cellulose approach using preactivators based on a cyanuric chloride core showed greater electronegativity for nucleophilic substitutions compared to other *N*-heterocyclic cores. These preactivators demonstrated symmetric substitutions that facilitated tandem reactions and eliminated negative cooperativity during functionalization. Our mechanistic approach involved the nonselective reaction of preactivators with the hydroxy groups of cellulose, followed by chemical-specific functionalization using the remaining two groups. The reactivity order of the preactivators with cellulose was PrAct1 (based on pyridine) > PrAct2 (based on N-methyl-imidazole) > PrAct3 (based on nicotinic acid), as observed in Figure 2a. Overall, our approach provides a promising pathway for the efficient functionalization of cellulose for various applications.

The two-step functionalization strategy was used for coloring cellulose fabric using two amine-containing colorants. The untreated cotton fabric sample did not absorb the dyes, while the preactivated cellulose fabrics absorbed the dyes considerably (Table 5). Such color testing experiments clearly proved the concept of a leaving group chemistry-based strategy for attaining the functionalization of cellulose materials. To expand the scope of the chemical functionalization of cellulose, we included peptides (cysteine and zein protein), non-aromatic hydrocarbons (1,5-pentane-diol, 1-amino-pentane, hexamine, octamine), and aromatic hydrocarbons (1-naphthol, benzylamine, benzyl alcohol, phenol). In addition, specific Raman bands and vibrational stretching were studied using time-gated Raman spectroscopy and FTIR spectroscopy for the representative class of functionalizing agents with respect to their functionalized cellulose material (which, in this case, was cotton fabric).

To understand the effects of leaving group chemistry on cellulose functionalization, we also synthesized four reactive chemicals based on the concept of leaving group chemistry. These reactive agents (Act1–4) were used for the functionalization of cellulose in a one-step reaction. The vibrational spectroscopy experiments showed a distinctive difference in the C-H stretching (in the case of non-aromatic hydrocarbons) and explicit aromatic regions (in the case of aromatic hydrocarbons) in the studied samples.

In conclusion, we developed a facile strategy for cellulose functionalization using a large series of application-oriented scaffolds (containing -NH_2_ or -OH groups), which can serve as a toolbox for surface chemical modifications onto cellulose. The cellulose functionalization was successfully achieved at room temperature without using corrosive alkaline salts (e.g., NaOH). This strategy can be potentially used for the functionalization of other substrates with hydroxy, carboxylic, or amine groups (e.g., wool, silk).

## Figures and Tables

**Table 1:** Reaction optimization for synthesis of Cellulose Preactivators.

Solvent	Additives (mol%)	*N*-Heterocycle	Temp.	Time (h)	Yields
Diethyl ether	-	Pyridine	r.t	24	Unreacted mixture
Ethyl acetate	-	Pyridine	Reflux	6	24
Cyclohexane	-	Pyridine	Reflux	4	Unreacted mixture
Acetone	-	Pyridine	Reflux	2	41
MeOH	-	Pyridine	Reflux	3	NR
CH_3_CN	-	Pyridine	Reflux	2	Inseparable mixture
THF	-	Pyridine	Reflux	3	36
DMF	-	Pyridine	Reflux	1	Inseparable mixture
DCM	-	Pyridine	r.t	8	*ni*
Ethyl acetate	Na_2_CO_3_ (0.5)	Pyridine	Reflux	4	43
Cyclohexane	Na_2_CO_3_(0.5)	Pyridine	Reflux	2	79
**Acetone**	**K_2_CO_3_(0.5)**	**Pyridine**	**Reflux**	**2**	**88**
Acetone	K_2_CO_3_(0.1)	Pyridine	Reflux	2	51
Acetone	K_2_CO_3_(1.0)	Pyridine	Reflux	2	85
Acetone	K_2_CO_3_(0.5)	Pyridine	r.t	2	42
Acetone	NaHCO_3_(0.5)	Pyridine	r.t	5	70
Acetone	NH_3_–MeOH (1.1)	Pyridine	reflux	1	Inseparable mixture
Acetone	NH_3_–MeOH (2.0)	Pyridine	r.t	4	Inseparable mixture

**Table 2:** Zeta potential measuring of preactivators onto the cellulose surface cationization. Optimization of preactivation-treated cellulose with functionalizing agents.

Type of Preactivators	Concentration (%*w*/*w*)	Solvent	Functionalizing Agent (10% *w*/*w*)	ζ-Potential (mV)
-	-	Acetone	-	−9.63
-	-	Acetone	choline chloride	−7.90
PrAct-1	10	Acetone	choline chloride	+15.53
PrAct-1	10	THF	choline chloride	+12.60
PrAct-1	10	DCM	choline chloride	+13.42
PrAct-1	10	CHCl_3_	choline chloride	+13.87
PrAct-1	10	Ethyl acetate	choline chloride	+11.55
PrAct-2	0.5	Acetone	choline chloride	+4.98
PrAct-2	1	Acetone	choline chloride	+7.25
PrAct-2	2.5	Acetone	choline chloride	+9.17
PrAct-2	5	Acetone	choline chloride	+10.04
PrAct-2	10	Acetone	choline chloride	+10.78
PrAct-3	0.5	Acetone	choline chloride	+4.43
PrAct-3	1	Acetone	choline chloride	+6.96
PrAct-3	2.5	Acetone	choline chloride	+8.79
PrAct-3	5	Acetone	choline chloride	+9.36
PrAct-3	10	Acetone	choline chloride	+10.02

**Table 3:** Effects of temperature on the chemical reactivity of choline chloride with preactivated cellulose with PrAct-1, PrAct-2, and PrAct-3.

Cellulose with Preactivators (10 wt.%)	ζ-Potential (mV)		
	Before Treating with Choline Chloride	After Treating with Choline Chloride (10% *w*/*w*)	
		r.t	T = 50°
PrAct-1	+11.38	+14.97	+18.19
PrAct-2	+7.60	+10.51	+12.70
PrAct-3	+3.95	+10.19	+11.57

**Table 4:** Coloration of preactivated cotton fabrics with two amine-containing colorants.

Colorant	PrAct-1	Sample Picture		Color Strength (K/S Values)		Color Fixation (%)
		Before Washing	After Washing	Before Washing	After Washing	
1	Yes	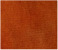	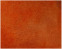	6.99	4.46	63.8
1	No	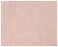	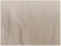	0.34	0.16	*No coloration*
2	Yes	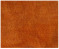	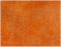	4.46	3.92	87.9
2	No	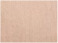	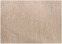	0.34	0.16	*No coloration*

**Table 5:** Addition of alkylating hydrocarbons.

Long-Chain Hydrocarbon	Modified Cellulose Form	FTIR Characterization
1,5-pentan-diol	*O*-tethering Pentoyl group	C-H stretching visible at 2913 cm^−1^, O-H stretching at 3341 cm^−1^
1-nonanol	*O*-tethering nonanyl group	C-H stretching visible at 2892 cm^−1^
Hexamine	*NH*-tethering Hexyl group	C-H stretching visible at 2952 cm^−1^, C-H stretching visible at 2952 cm^−1^, -H stretching at 3419 cm^−1^
Octamine	*NH*-tethering Octyl group	Sharp C-H stretching visible at 2968 cm^−1^, N-H stretching at 3408 cm^−1^
**Aromatic hydrocarbons**		
1-napthol	*O*-tethering naptholyl group	Aromatic region stretching visible 1340–1480 cm^−1^
Benzylamine	*NH*-tethering Benzyl group	C-H stretching visible at 2952 cm^−1^ N-H stretching at 3419 cm^−1^
Benzyl alcohol	*O*-tethering Benzyl group	Aromatic region stretching visible at 1390–1460 cm^−1^ O-H stretching at 3341 cm^−1^
Phenol	*O*-tethering Phenyl group	Aromatic region stretching visible at 1380–1450 cm^−1^

## Data Availability

The data used in this research paper are available for access and reuse.

## Supplementary Materials

The following supporting information can be downloaded at: https://www.mdpi.com/article/10.3390/polym16010149/s1, Figure S1: 1H-NMR of 1,1′,1″-(1,3,5-triazine-2,4,6-triyl)tris(pyridin-1-ium) (PrAct-1); Figure S2: 13C-NMR of 1,1′,1″-(1,3,5-triazine-2,4,6-triyl)tris(pyridin-1-ium) (PrAct-1); Figure S3: 1H-NMR of 3,3′,3″-(1,3,5-triazine-2,4,6-triyl)tris(1-methyl-1H-imidazol-3-ium) (PrAct-2); Figure S4: 13C-NMR of 3,3′,3″-(1,3,5-triazine-2,4,6-triyl)tris(1-methyl-1H-imidazol-3-ium) (PrAct-2); Figure S5: 1H-NMR of 1,1′,1″-(1,3,5-triazine-2,4,6-triyl)tris(3-carboxypyridin-1-ium) (PrAct-3); Figure S6: 13C-NMR of 1,1′,1″-(1,3,5-triazine-2,4,6-triyl)tris(3-carboxypyridin-1-ium) (PrAct-3); Figure S7: 1H-NMR of 2-pyridinium methyl phenyl ketone (Act-1); Figure S8: 1H-NMR of 2-pyridinium methyl decanyl ketone (Act-2); Figure S9: 1H-NMR of 3-carboxy-1-dodecylpyridin-1-ium (Act-3); Figure S10: 1H-NMR of 2-nicotinyl methyl phenyl ketone (Act-4); Figure S11: Reflectance spectroscopical data for colorant-1; Figure S12: Reflectance spectroscopical data for colorant-2; Figure S13 FTIR Spectroscopy: Cysteine-treated preactivated cotton fabrics; Figure S14 FTIR Spectroscopy: Zein-treated preactivated cotton fabrics; Figure S15: Time-Gated Raman spectroscopy: 1-Nonanol-treated preactivated cotton fabrics; Figure S16: Time-Gated Raman spectroscopy: 1,8-Octadiol-treated preactivated cotton fabrics; Figure S17: Time-Gated Raman spectroscopy: Cysteine-treated preactivated cotton fabrics; Figure S18: Time-Gated Raman spectroscopy: Benzylamine-treated preactivated cotton fabrics; Figure S19: Time-Gated Raman spectroscopy: Phenol-treated preactivated cotton fabrics; Figure S20: Time-Gated Raman spectroscopy: Act-1-treated cotton fabrics; Figure S21: Time-Gated Raman spectroscopy: Act-2-treated cotton fabrics.
