# Supplementary material for: Cellulose Functionalization Using N-Heterocyclic-Based Leaving Group Chemistry
Source: Polymers (Basel). 2024 Jan 3;16(1):149. doi: 10.3390/polym16010149 (PMC10780667; doi:10.3390/polym16010149)

Supporting Information for

Cellulose Functionalization Based on *N*-Heterocyclic based Leaving Group Chemistry

Arvind Negi, Ali R. Tehrani-Bagha [*]

Department of Bioproducts and Biosystems, School of Chemical Engineering, Aalto University, 02150 Espoo, Finland

[*]*Corresponding author: ali.tehrani@aalto.fi*

Contents

Figures	***NMR assignments of representative compounds***
S1	^{1}H-NMR of *1,1',1''-(1,3,5-triazine-2,4,6-triyl)tris(pyridin-1-ium)* (PrAct-1)
S2	^{13}C- NMR of *1,1',1''-(1,3,5-triazine-2,4,6-triyl)tris(pyridin-1-ium)* (PrAct-1)
S3	^{1}H-NMR of *3,3',3''-(1,3,5-triazine-2,4,6-triyl)tris(1-methyl-1H-imidazol-3-ium)* (PrAct-2)
S4	^{13}C-NMR of *3,3',3''-(1,3,5-triazine-2,4,6-triyl)tris(1-methyl-1H-imidazol-3-ium)* (PrAct-2)
S5	^{1}H-NMR of *1,1',1''-(1,3,5-triazine-2,4,6-triyl)tris(3-carboxypyridin-1-ium)* (PrAct-3)
S6	^{13}C-NMR of *1,1',1''-(1,3,5-triazine-2,4,6-triyl)tris(3-carboxypyridin-1-ium)* (PrAct-3)
S7	^{1}H-NMR of *2-pyridinium methyl phenyl ketone* (Act-1)
S8	^{1}H-NMR of *2-pyridinium methyl decanyl ketone* (Act-2)
S9	^{1}H-NMR of *3-carboxy-1-dodecylpyridin-1-ium* (Act-3)
S10	^{1}H-NMR of *2-nicotinyl methyl phenyl ketone* (Act-4)
	Reflectance Spectroscopy: Results for anthraquinone based colorants
S11	Reflectance spectroscopical data for colorant-1
S12	Reflectance spectroscopical data for colorant-2
	FTIR Spectroscopy: Chemical characterization of cellulose functionalisation
S13	Cysteine-treated preactivated cotton fabrics
S14	Zein-treated preactivated cotton fabrics
	Time-Gated Raman spectroscopy: Chemical characterization of cellulose functionalisation
S15	1-Nonanol-treated preactivated cotton fabrics
S16	1,8-Octadiol-treated preactivated cotton fabrics
S17	Cysteine-treated preactivated cotton fabrics
S18	Benzylamine-treated preactivated cotton fabrics
S19	Phenol-treated preactivated cotton fabrics
S20	Act-1-treated cotton fabrics
**S21**	Act-2-treated cotton fabrics

S1. ^{1}H-NMR of *1,1',1''-(1,3,5-triazine-2,4,6-triyl)tris(pyridin-1-ium)* (PrAct-1)

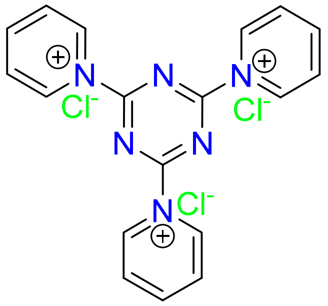

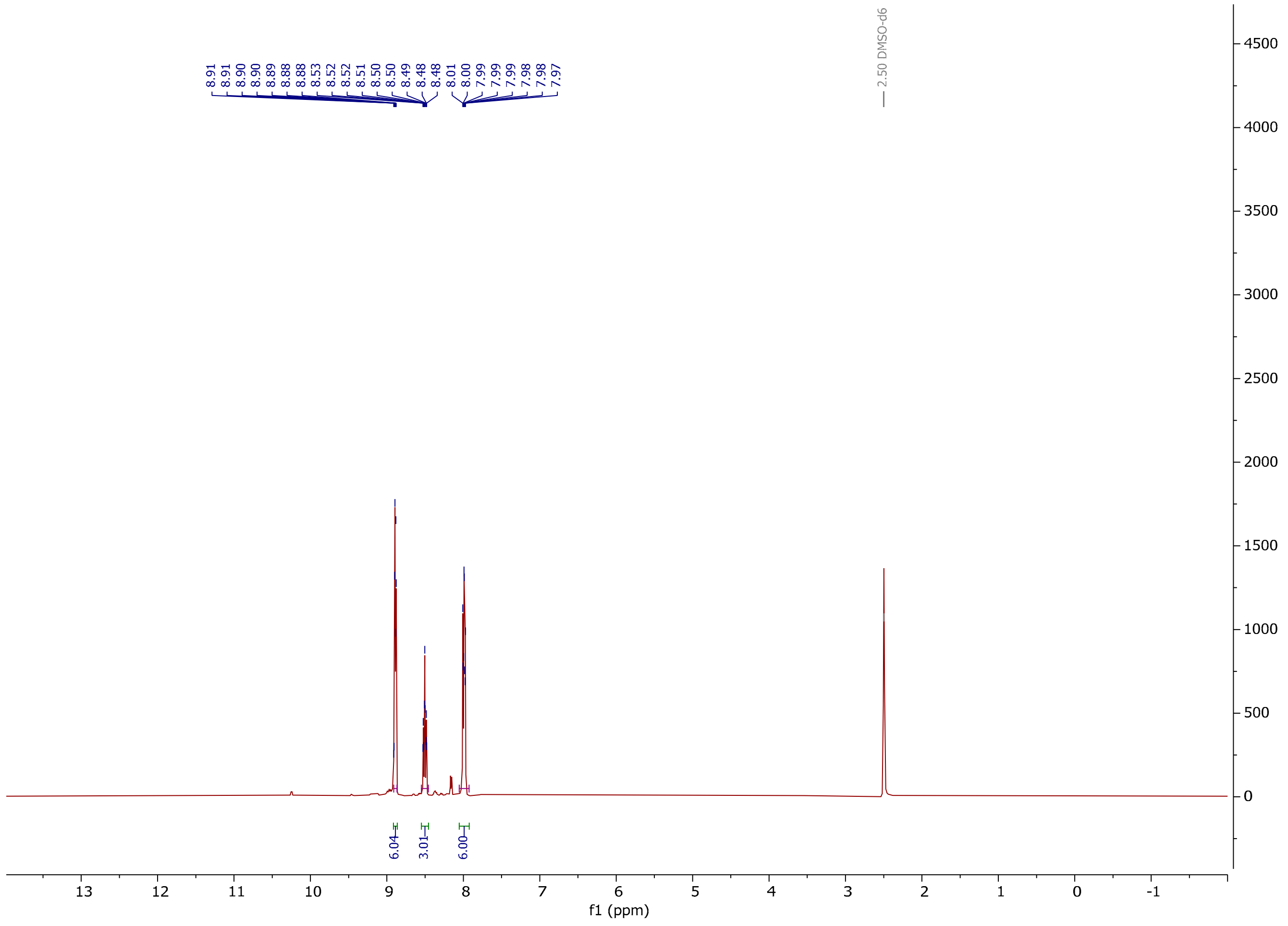

S2. ^{13}C-NMR of *1,1',1''-(1,3,5-triazine-2,4,6-triyl)tris(pyridin-1-ium)* (PrAct-1)

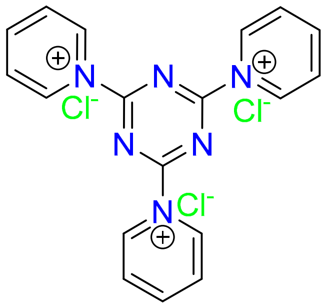

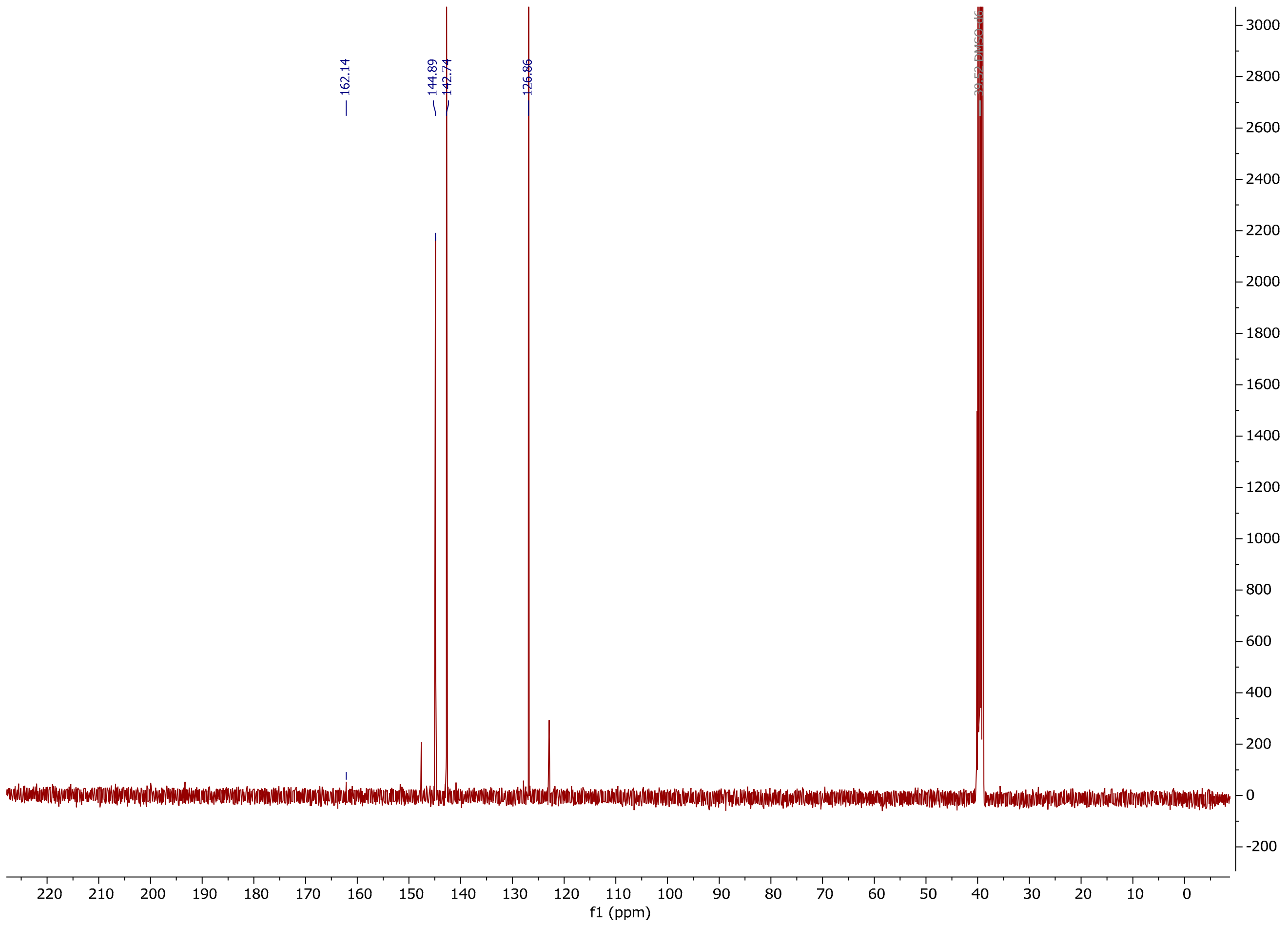

S3. ^{1}H-NMR of *3,3',3''-(1,3,5-triazine-2,4,6-triyl)tris(1-methyl-1H-imidazol-3-ium)* (PrAct-2)

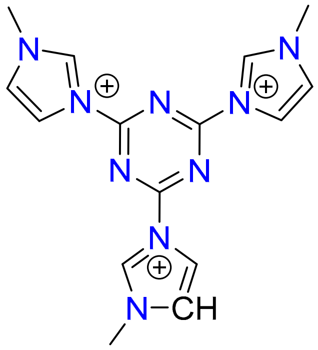

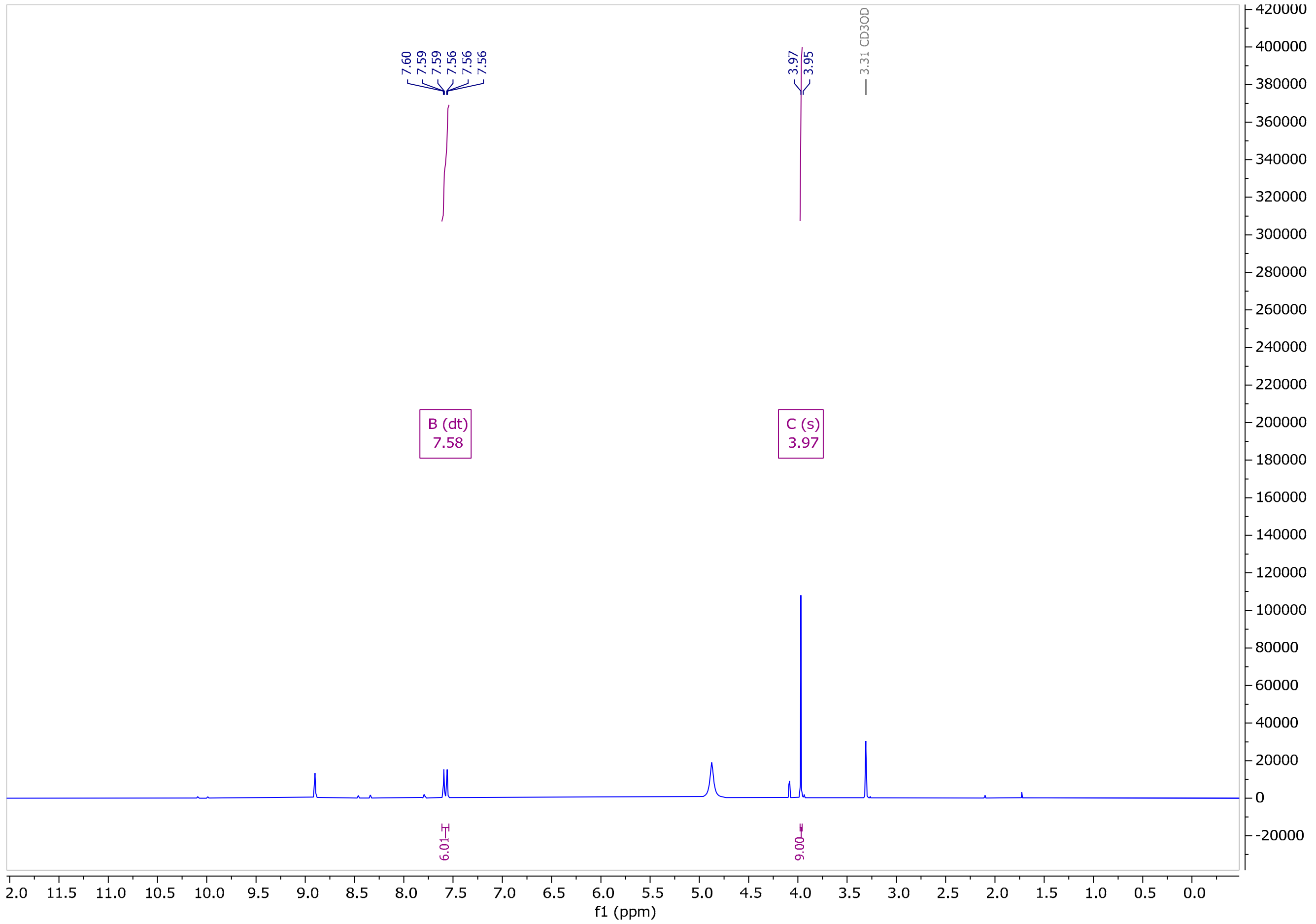

S4. ^{1}H-NMR of *3,3',3''-(1,3,5-triazine-2,4,6-triyl)tris(1-methyl-1H-imidazol-3-ium)* (PrAct-2)

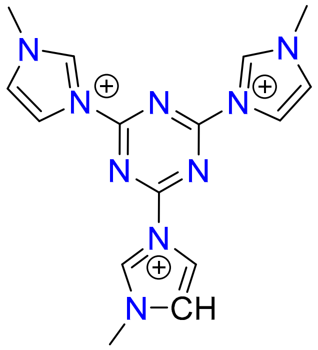

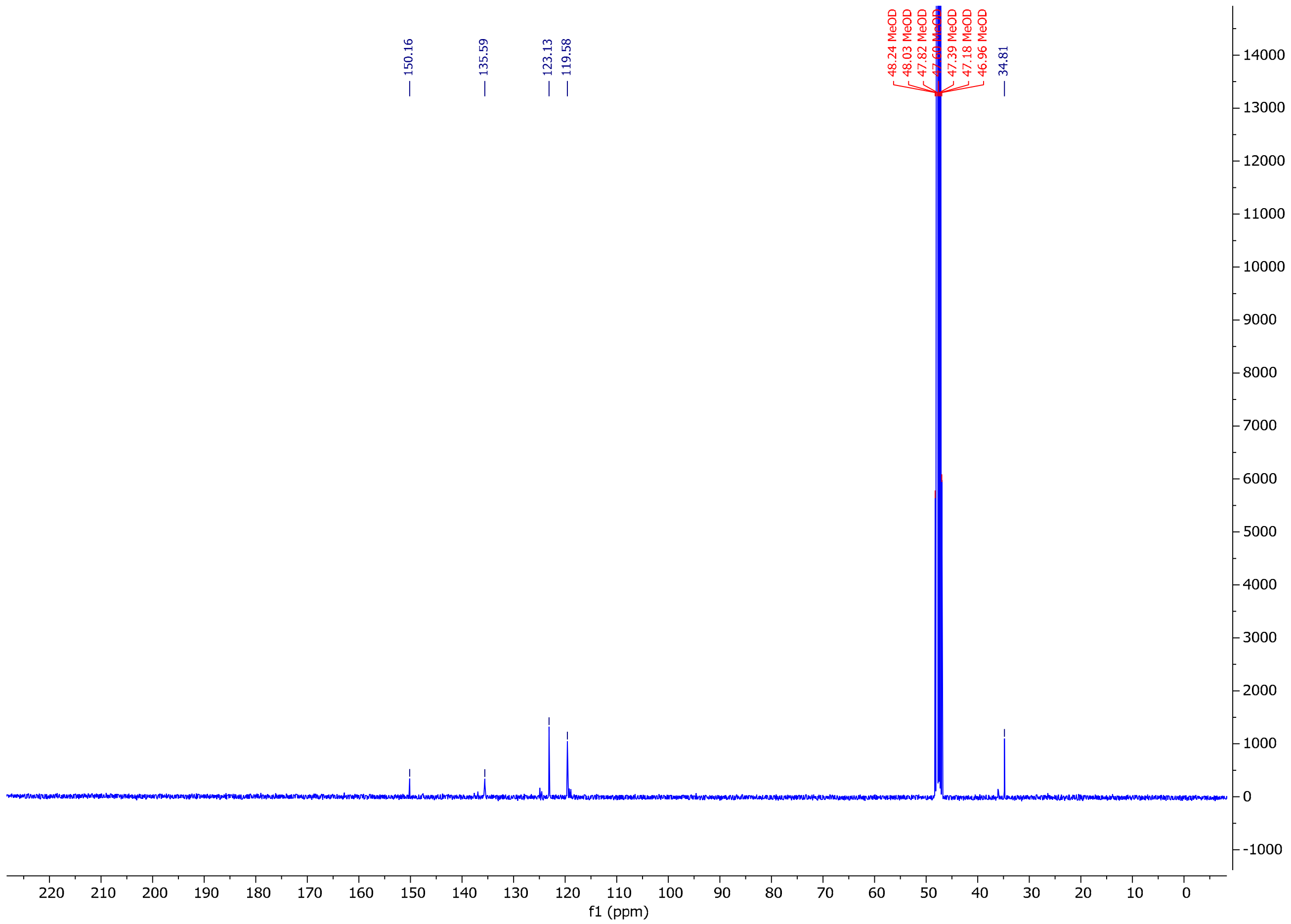

S5. ^{1}H-NMR of *1,1',1''-(1,3,5-triazine-2,4,6-triyl)tris(3-carboxypyridin-1-ium)* (PrAct-3)

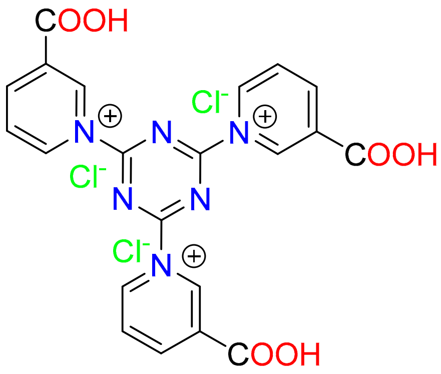

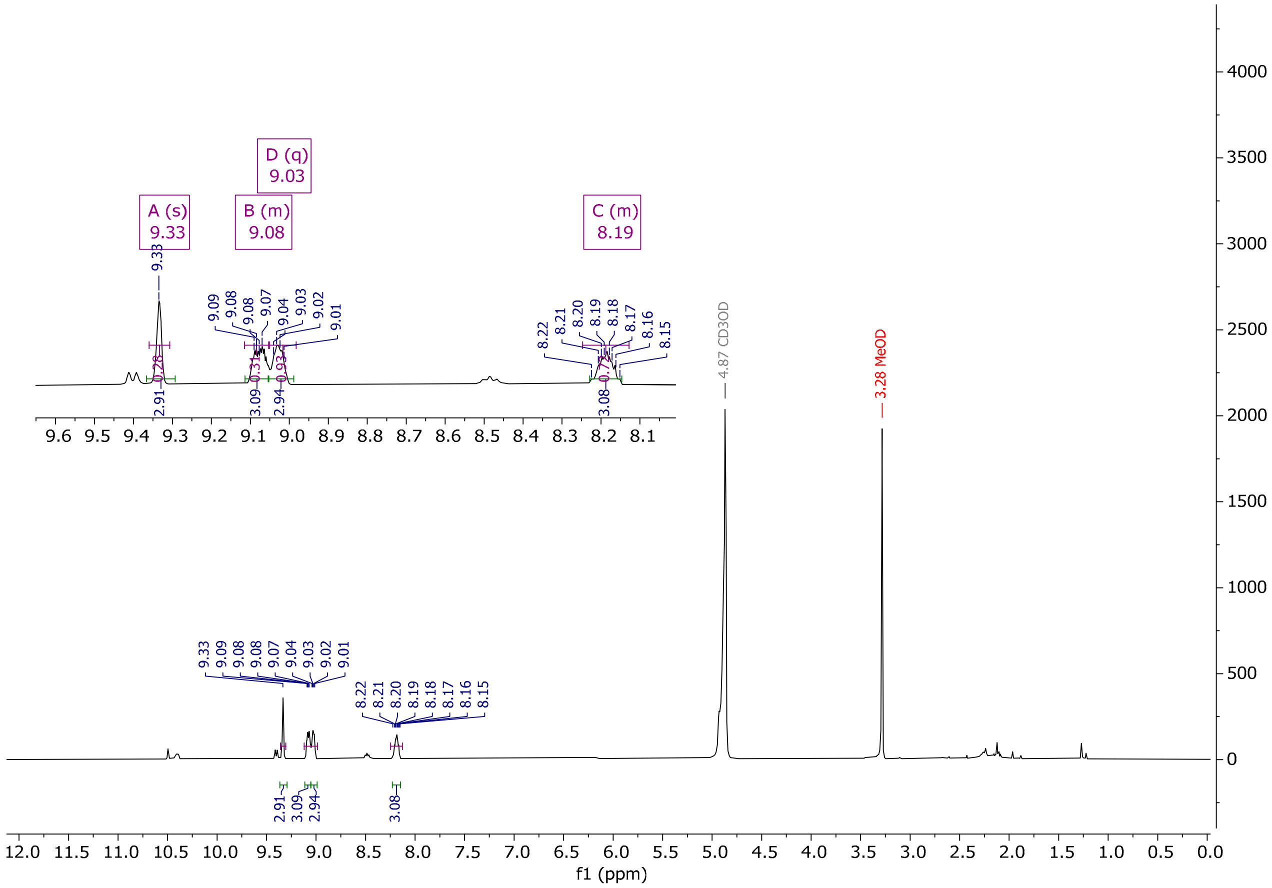

S6. ^{13}C-NMR of *1,1',1''-(1,3,5-triazine-2,4,6-triyl)tris(3-carboxypyridin-1-ium)* (PrAct-3)

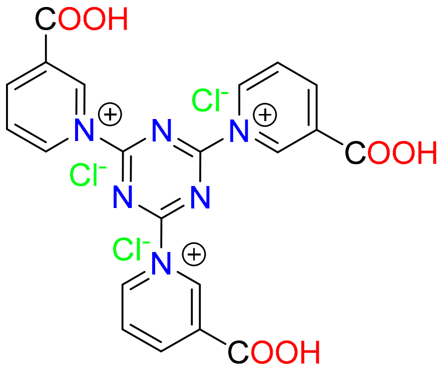

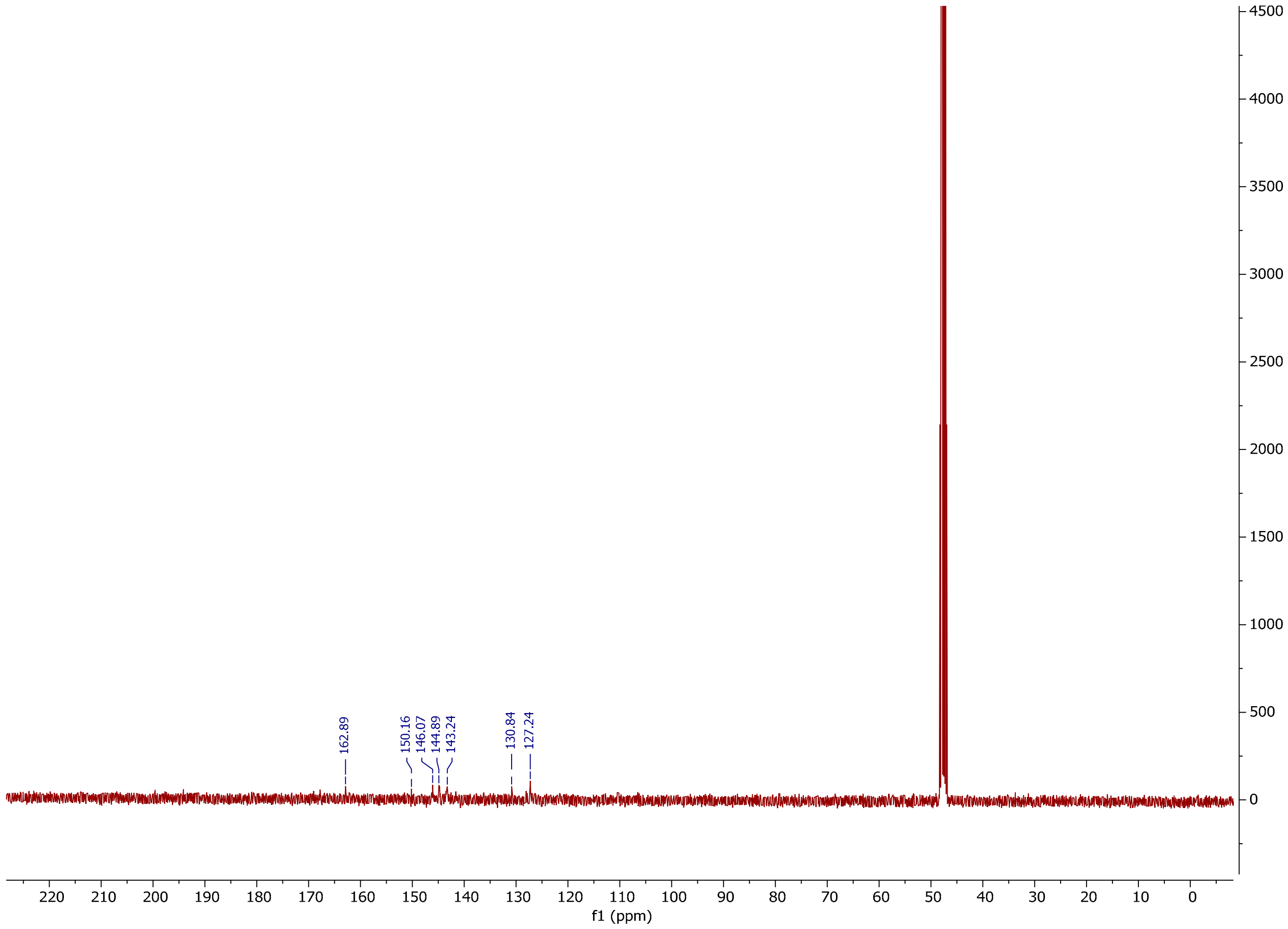

S7. ^{1}H-NMR of *2-pyridinium methyl phenyl ketone* (Act-1)

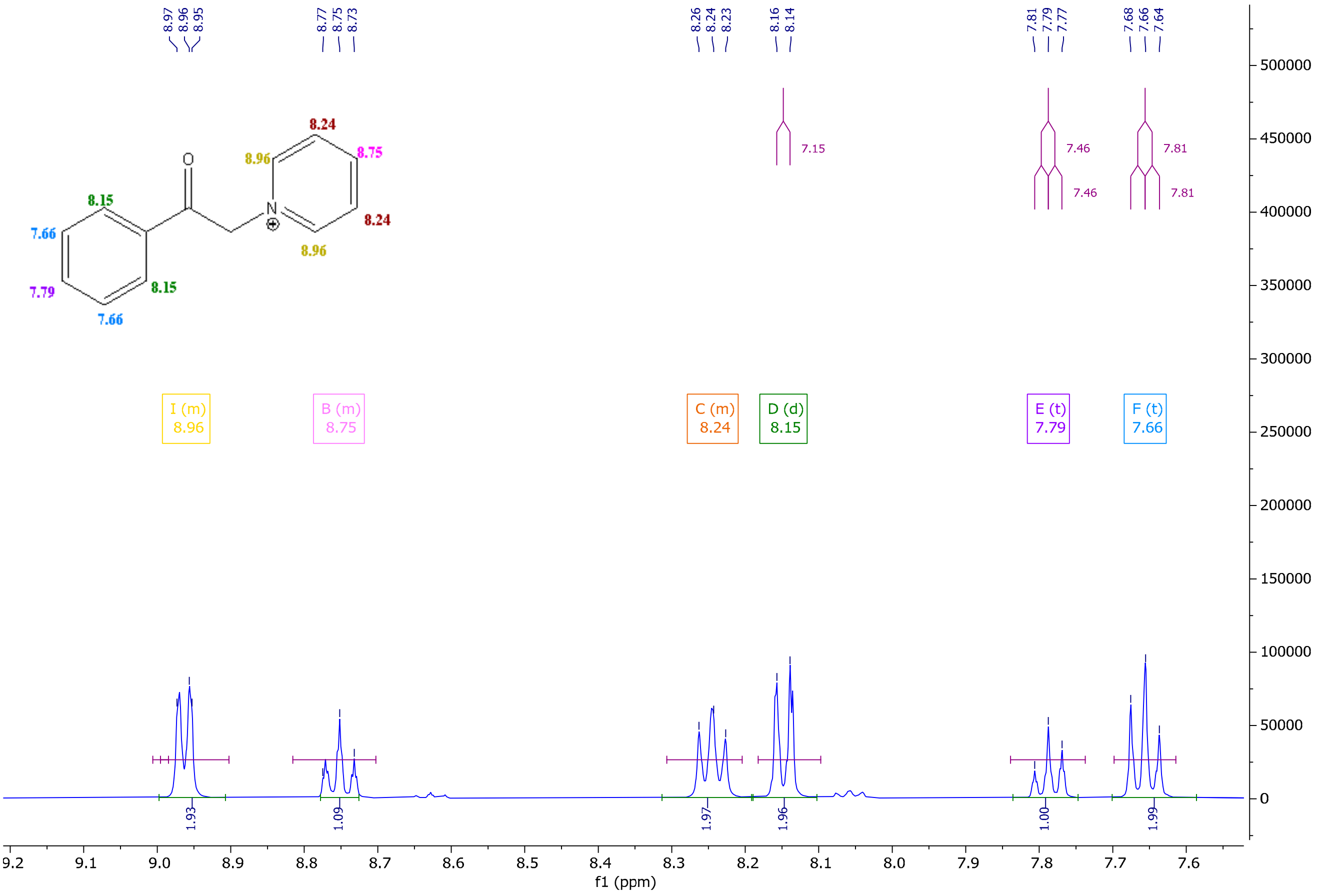

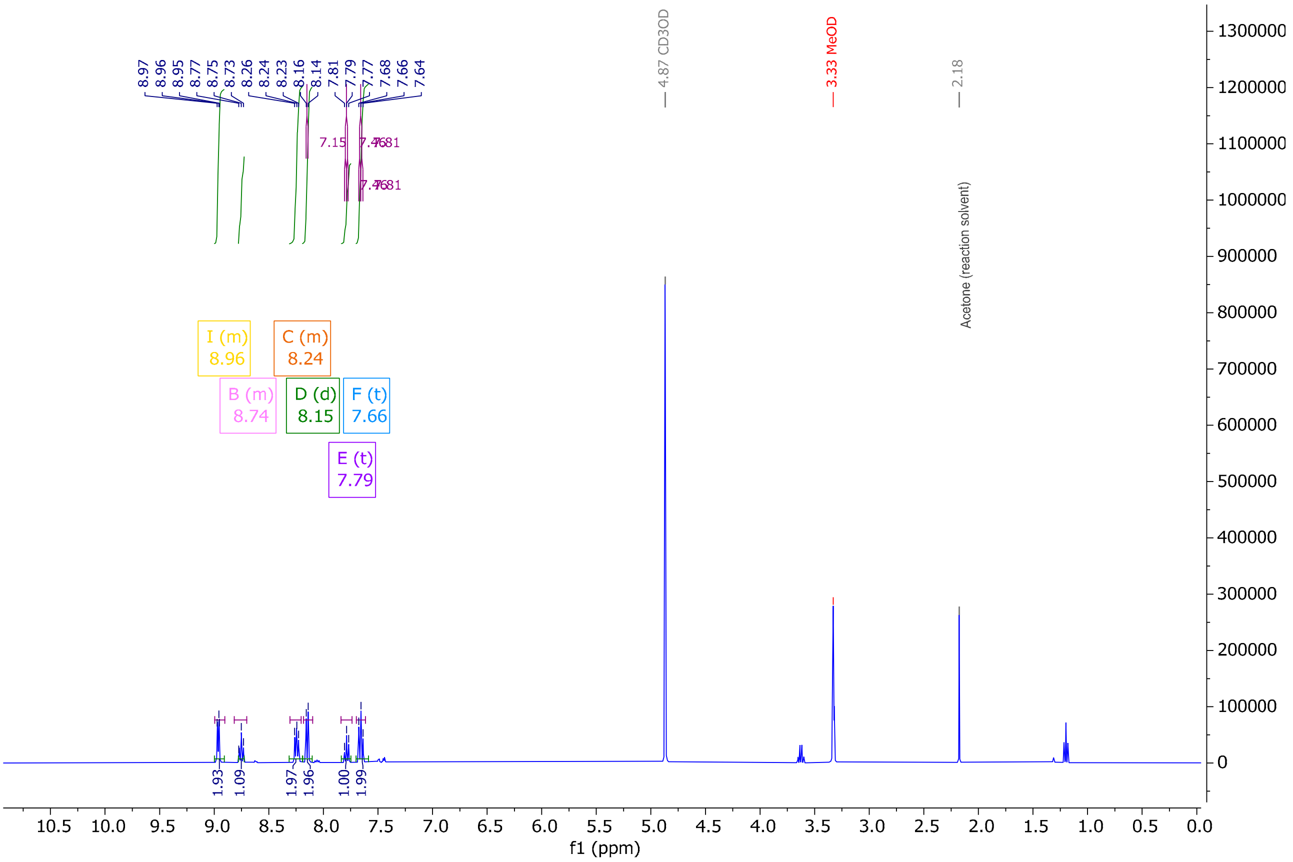

S8. ^{1}H-NMR of *2-pyridinium methyl decanyl ketone* (Act-2)

^{1}H-NMR in CD_3OD

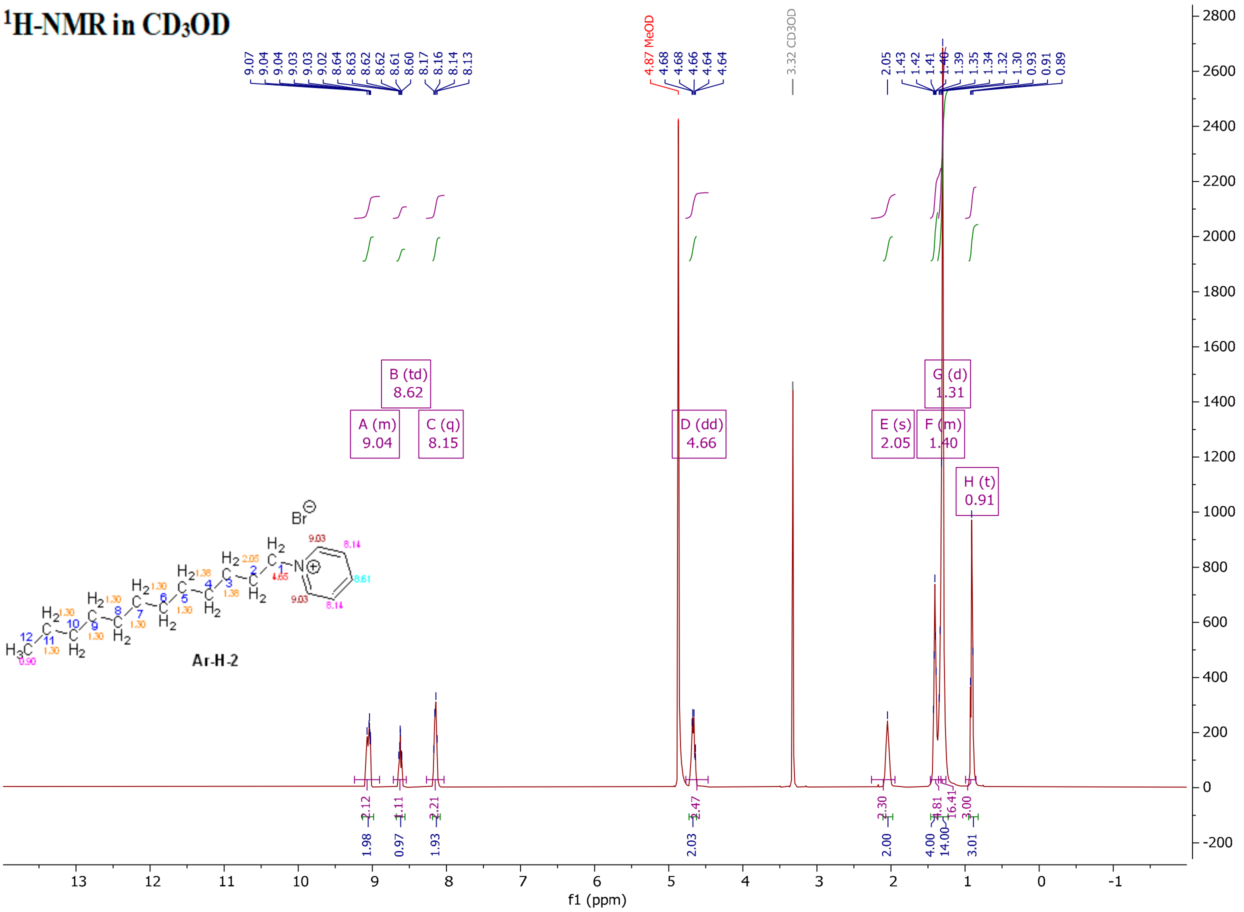

^{1}H-NMR in DMSO-*D6*

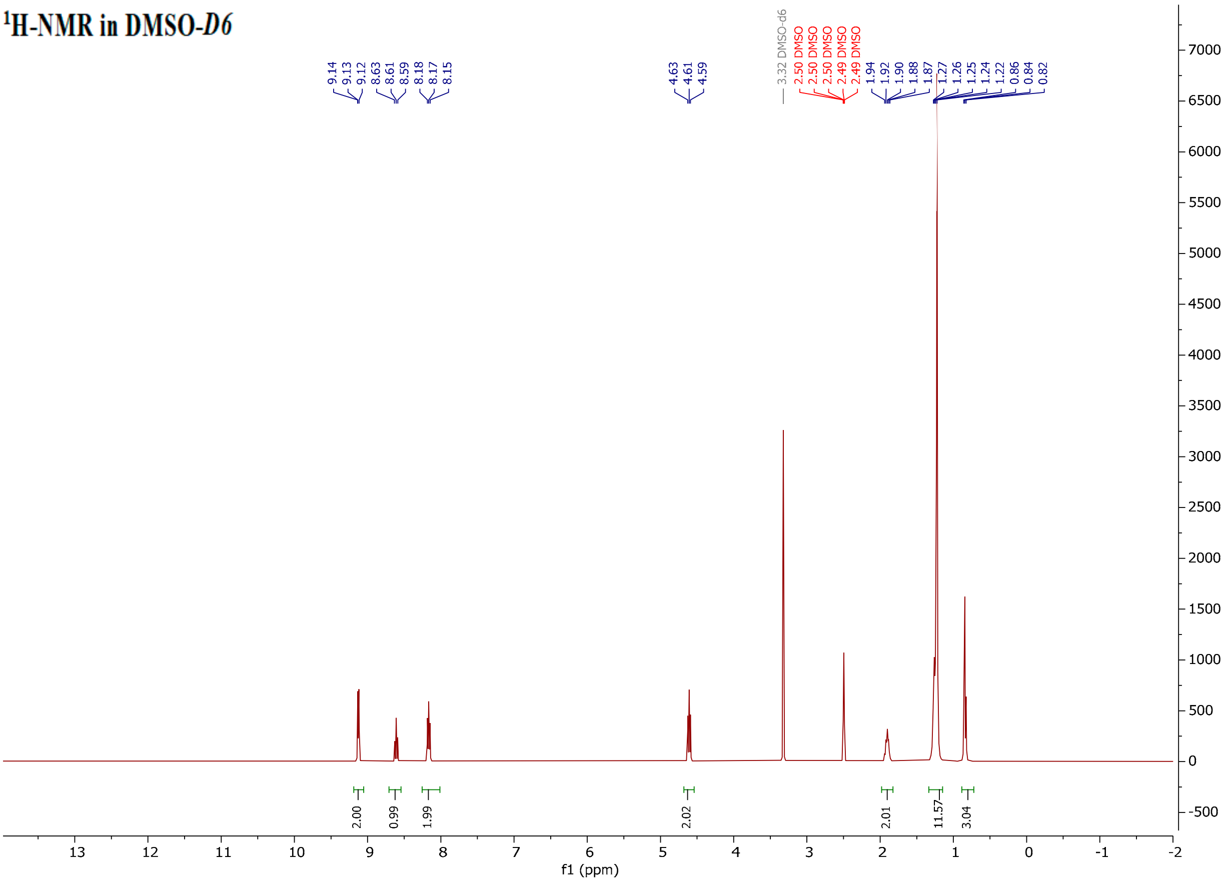

S9. ^{1}H-NMR of *3-carboxy-1-dodecylpyridin-1-ium* (Act-3)

^{1}H-NMR in DMSO-*D6*

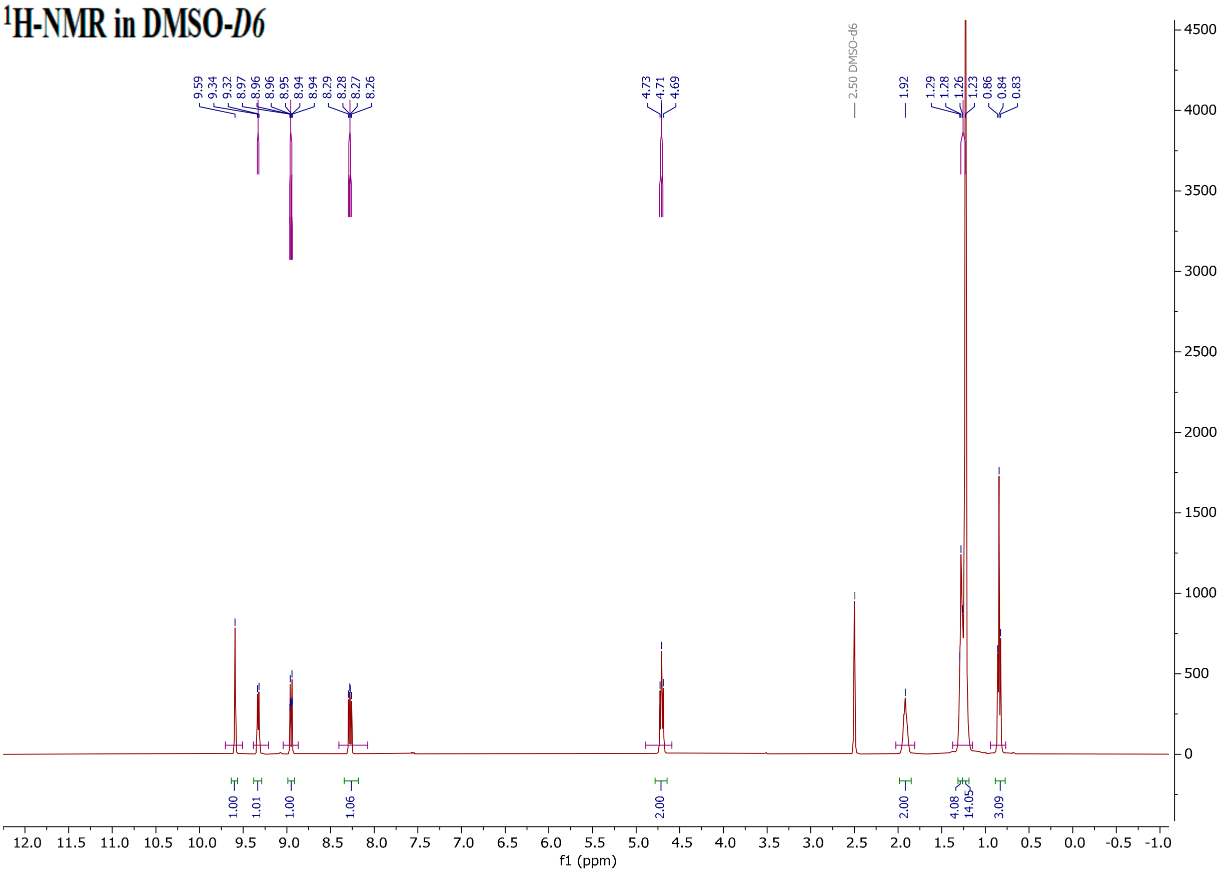

^{1}H-NMR in CD_3OD

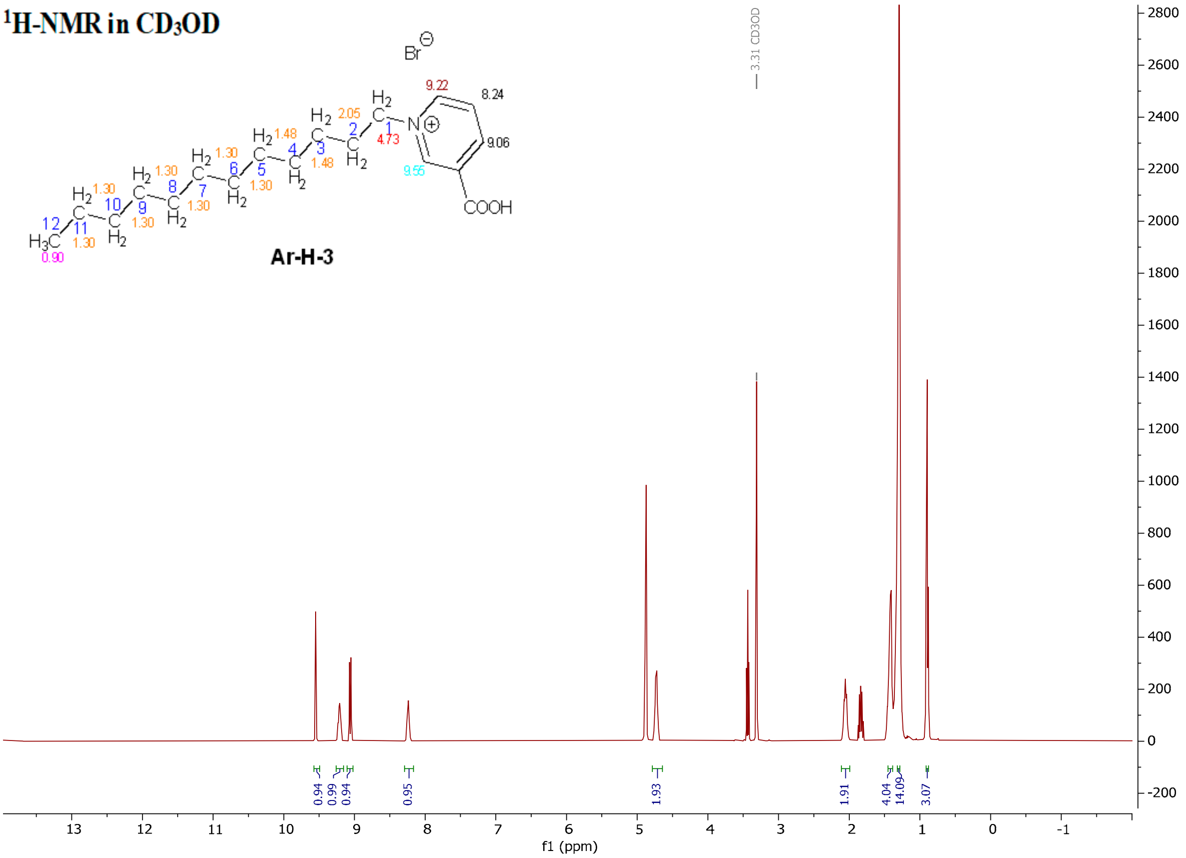

S10. ^{1}H-NMR of *2-nicotinyl methyl phenyl ketone* (Act-4)

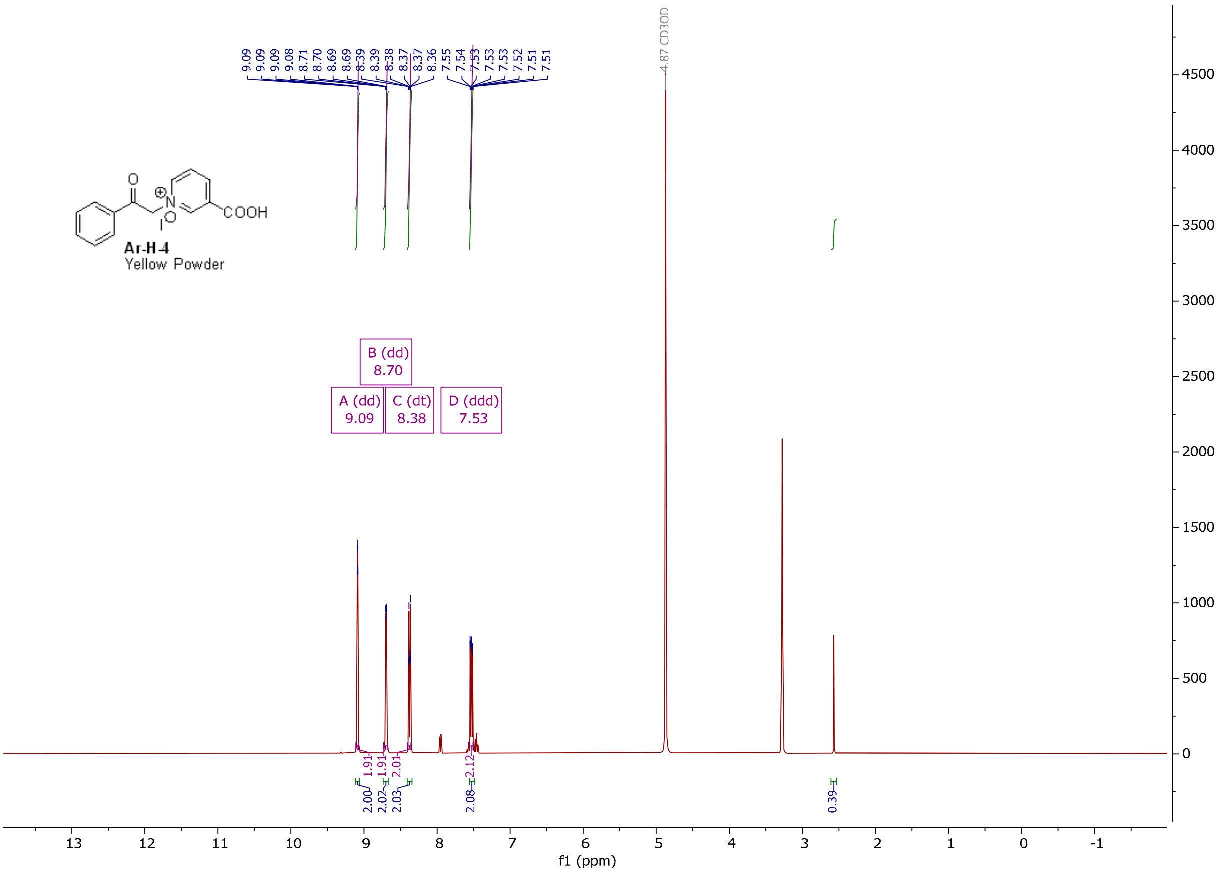

S11. Reflectance spectroscopical data for colorant-1 on PrAct-1 preactivated and nonactivated cellulose.

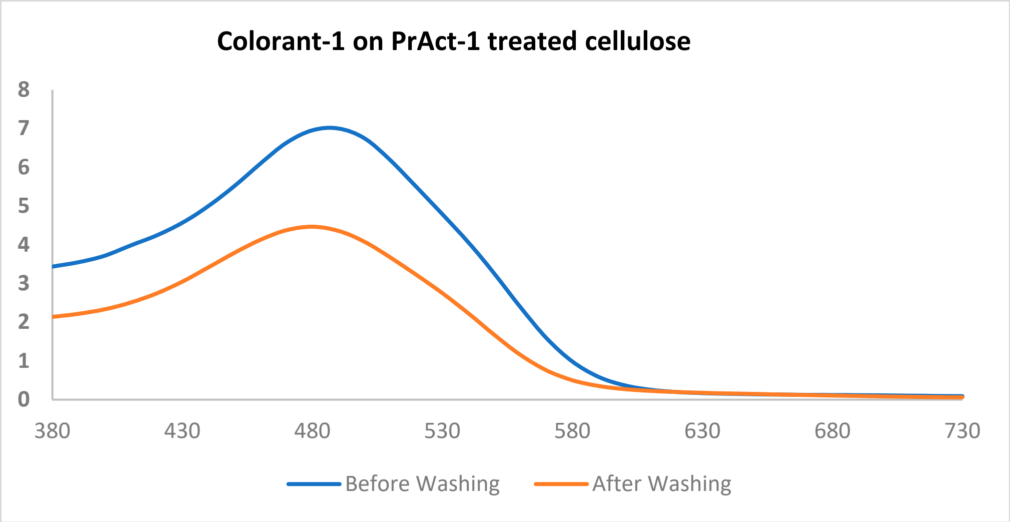

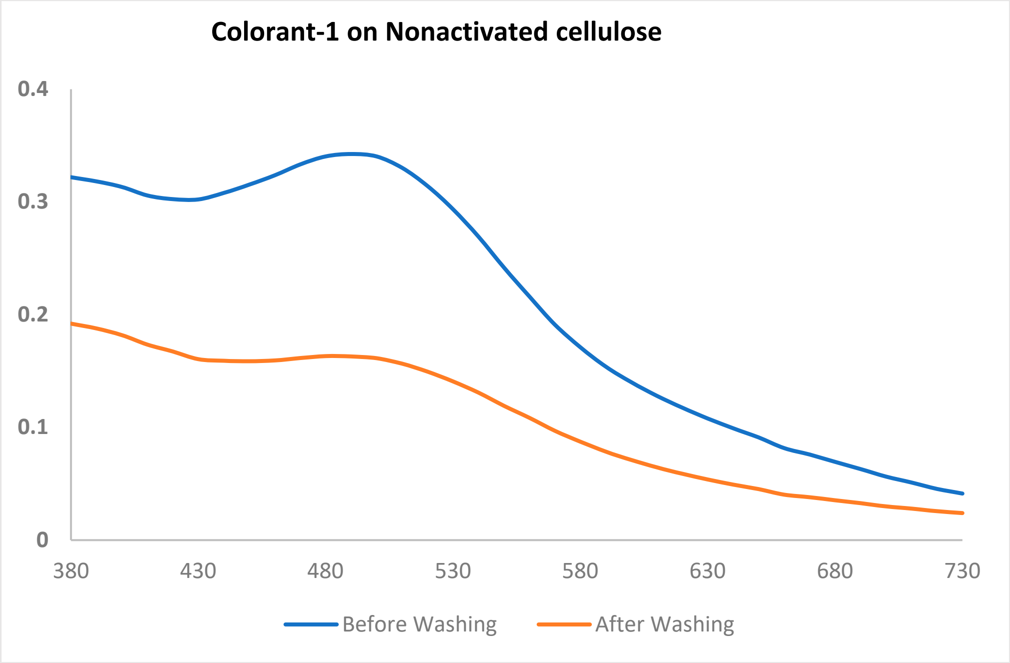

S12. Reflectance spectroscopical data for colorant-2 on PrAct-1 preactivated and nonactivated cellulose.

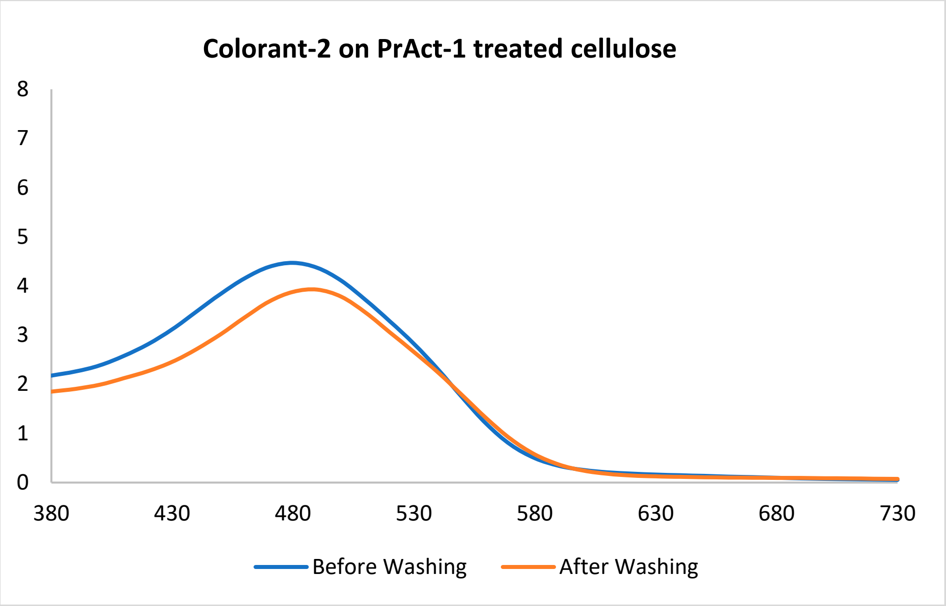

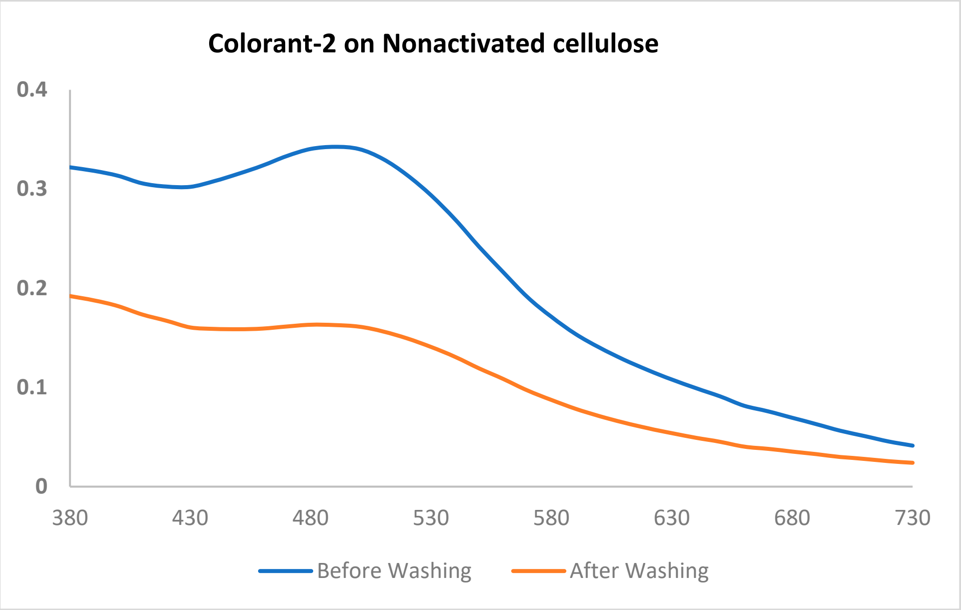

S13. FTIR Spectroscopy: Chemical characterization of Cysteine-treated preactivated cotton fabrics

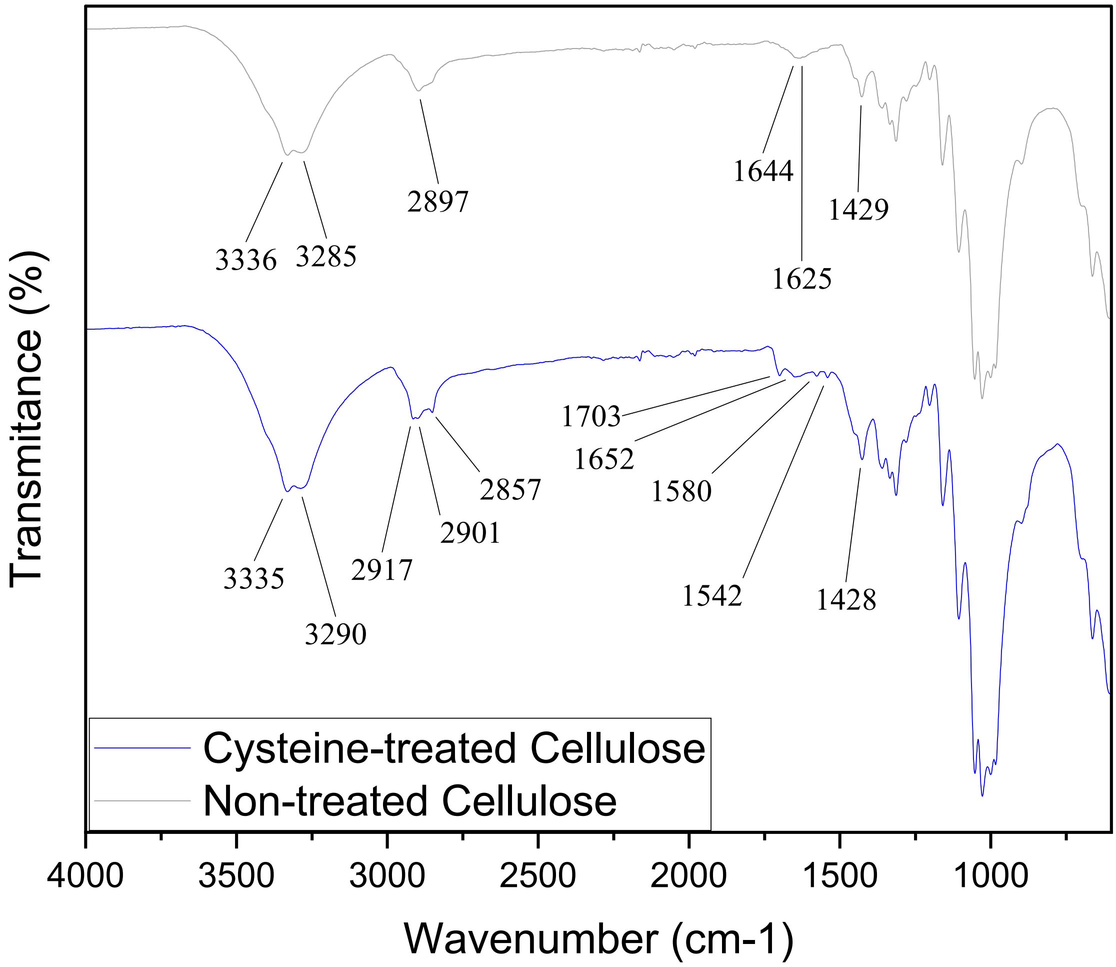

S14. FTIR Spectroscopy: Chemical characterization of Zein-treated preactivated cotton fabrics

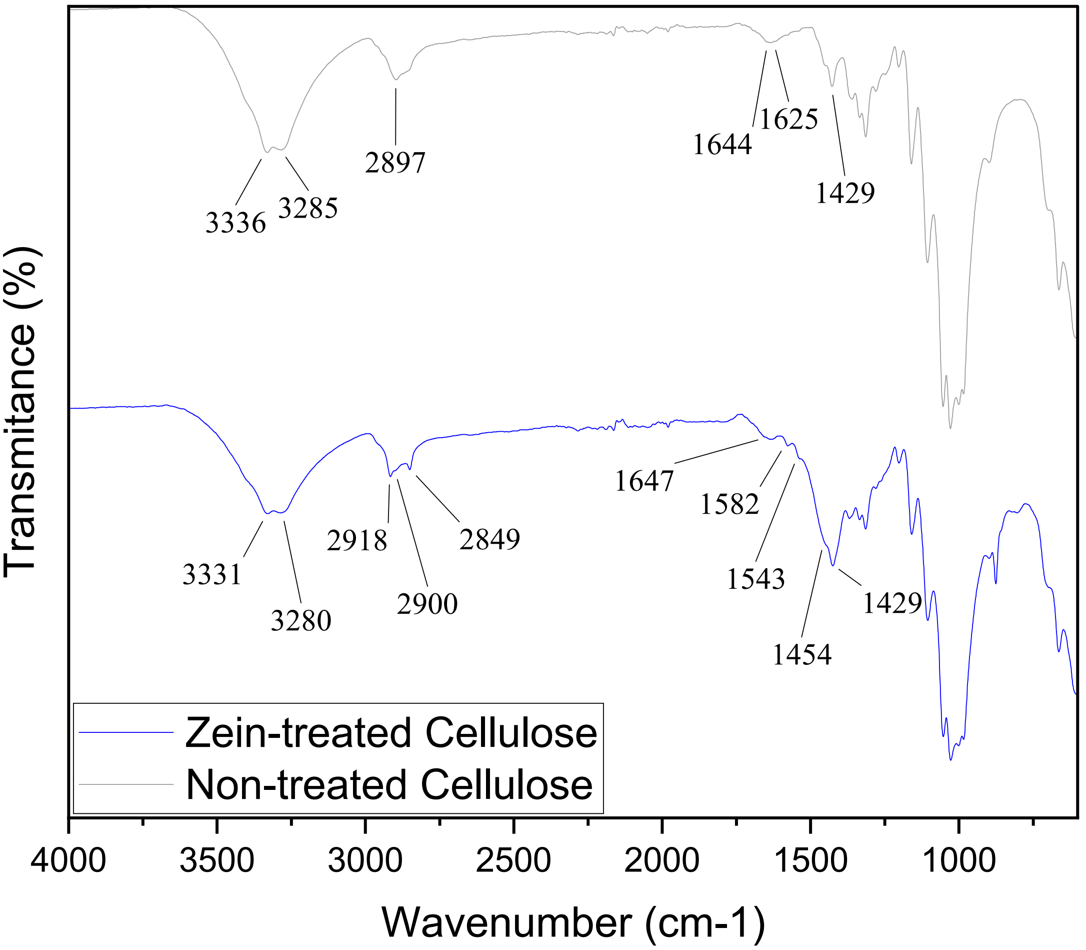

S15 1-Nonanol-treated preactivated cotton fabrics

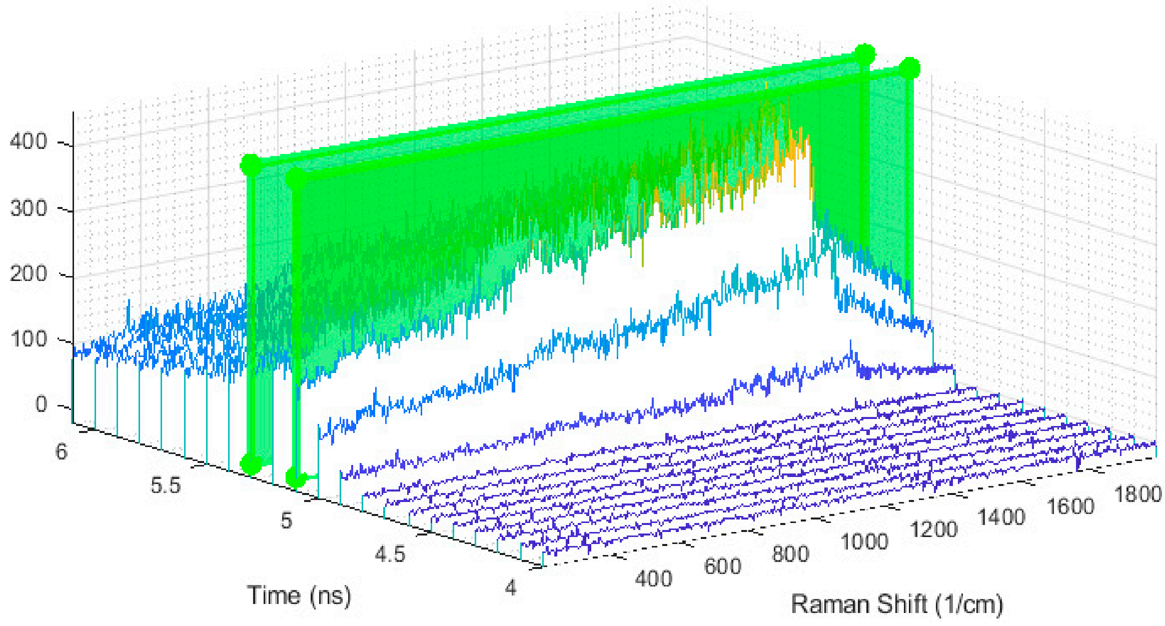

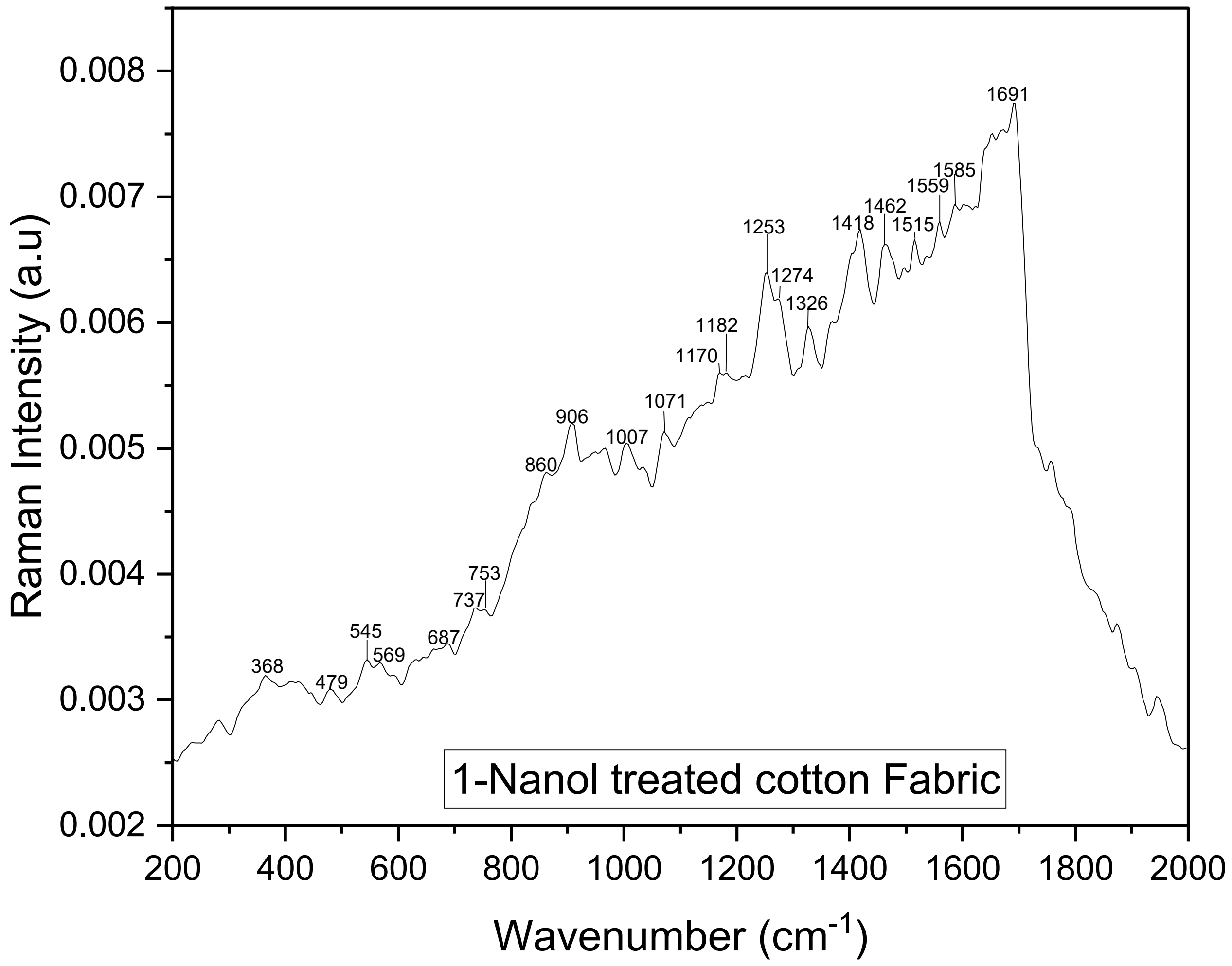

S16 1,8-Octadiol-treated preactivated cotton fabrics

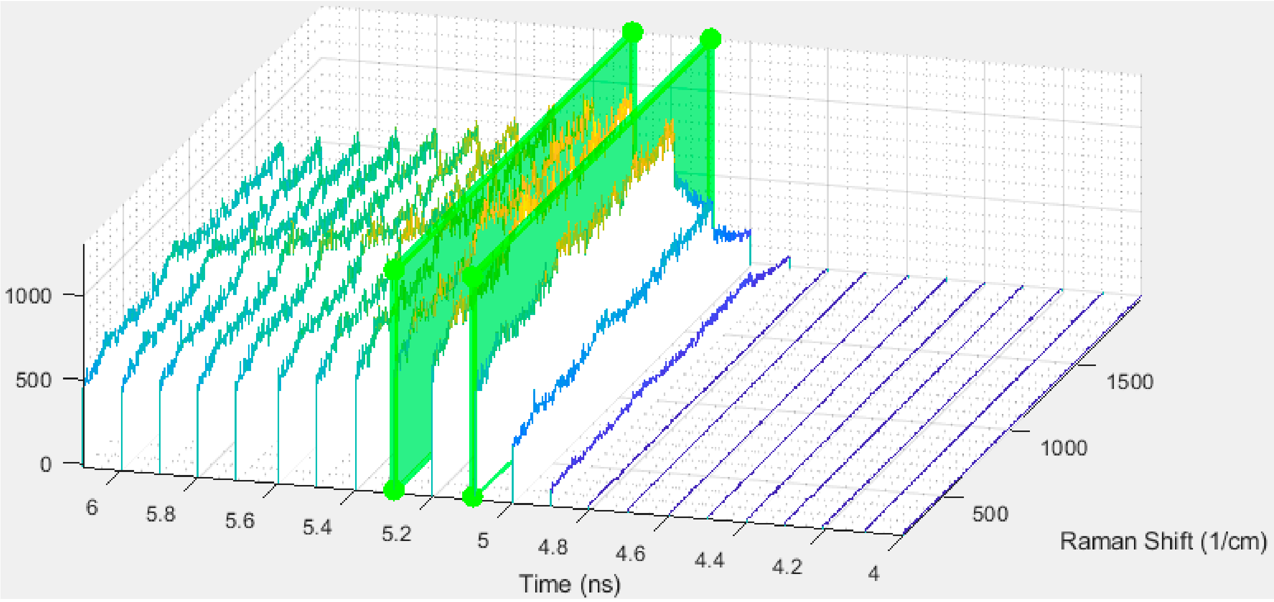

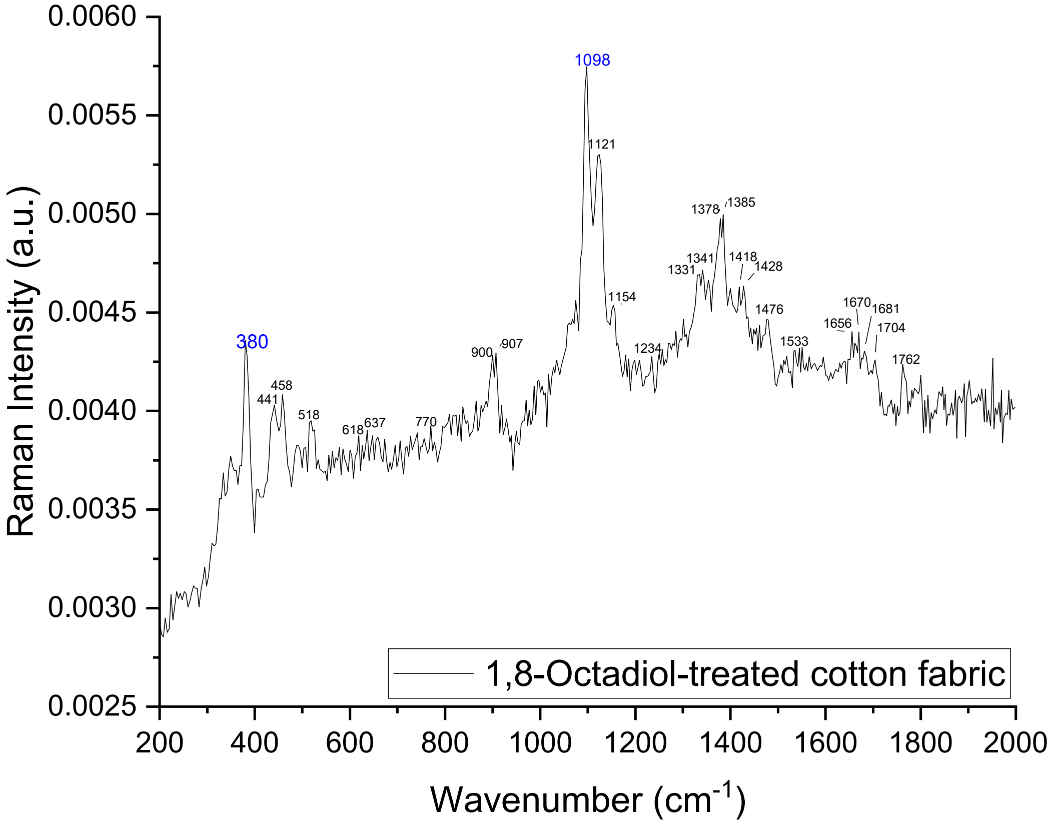

S17 Cysteine-treated preactivated cotton fabrics

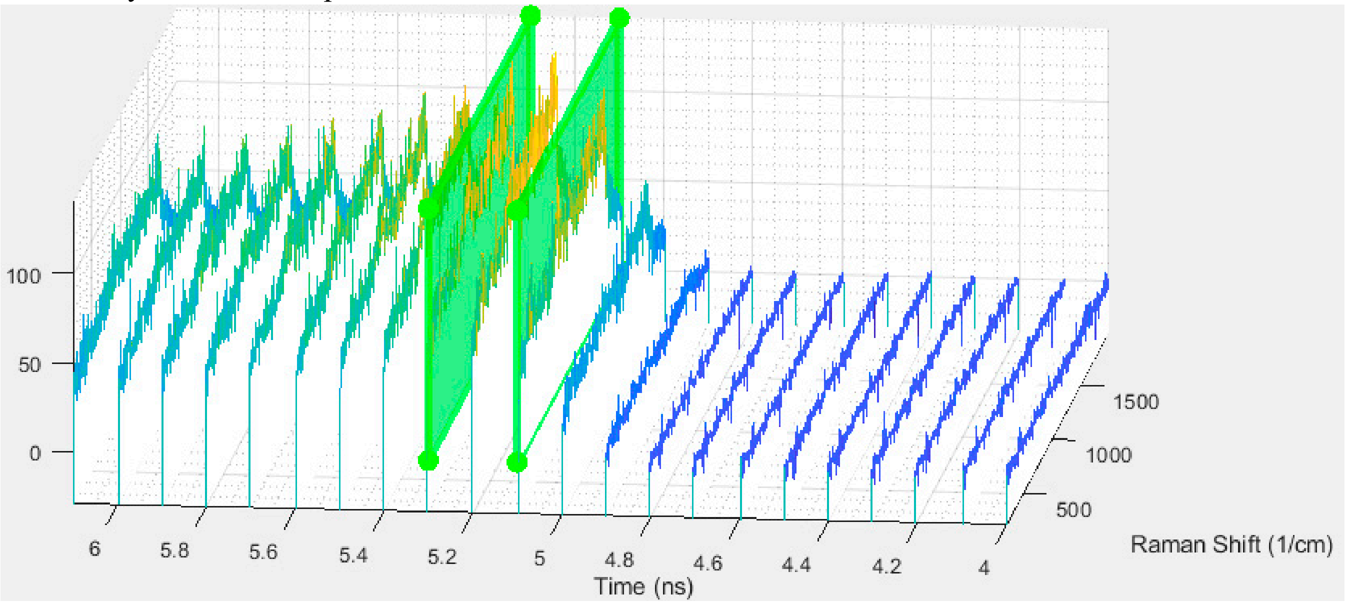

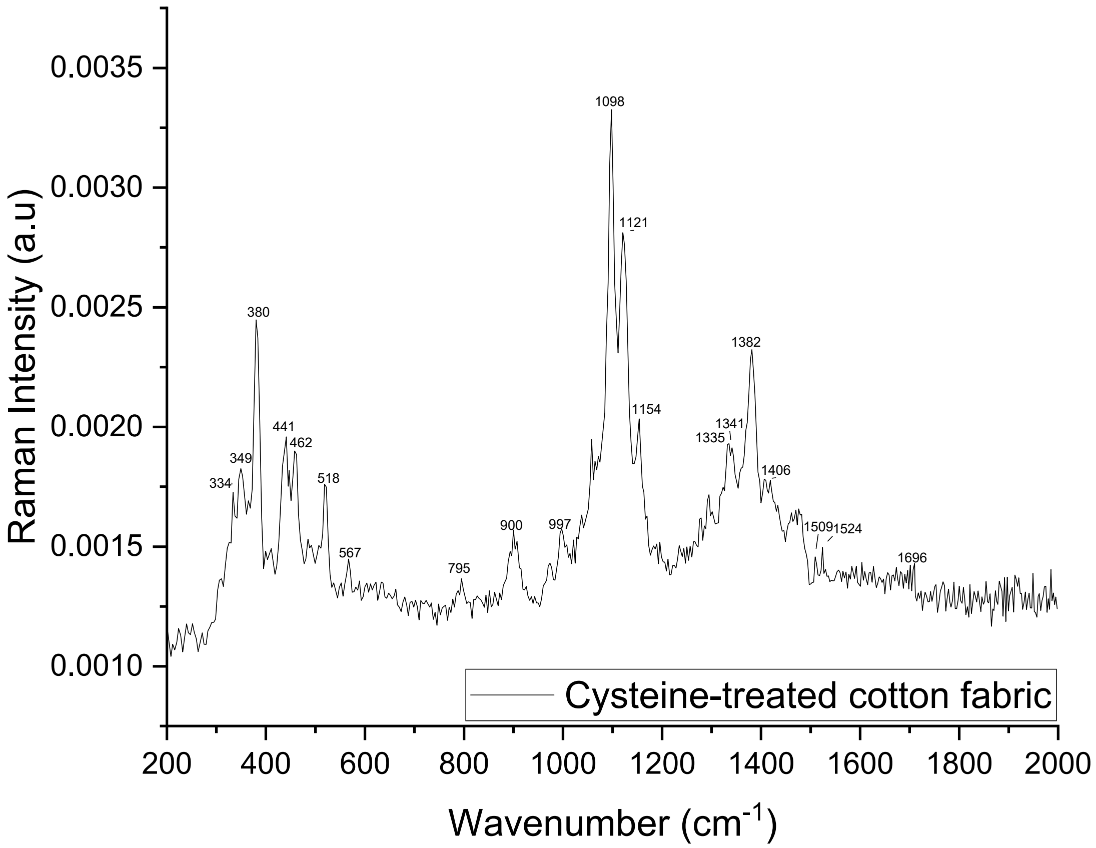

S18 Benzylamine-treated preactivated cotton fabrics

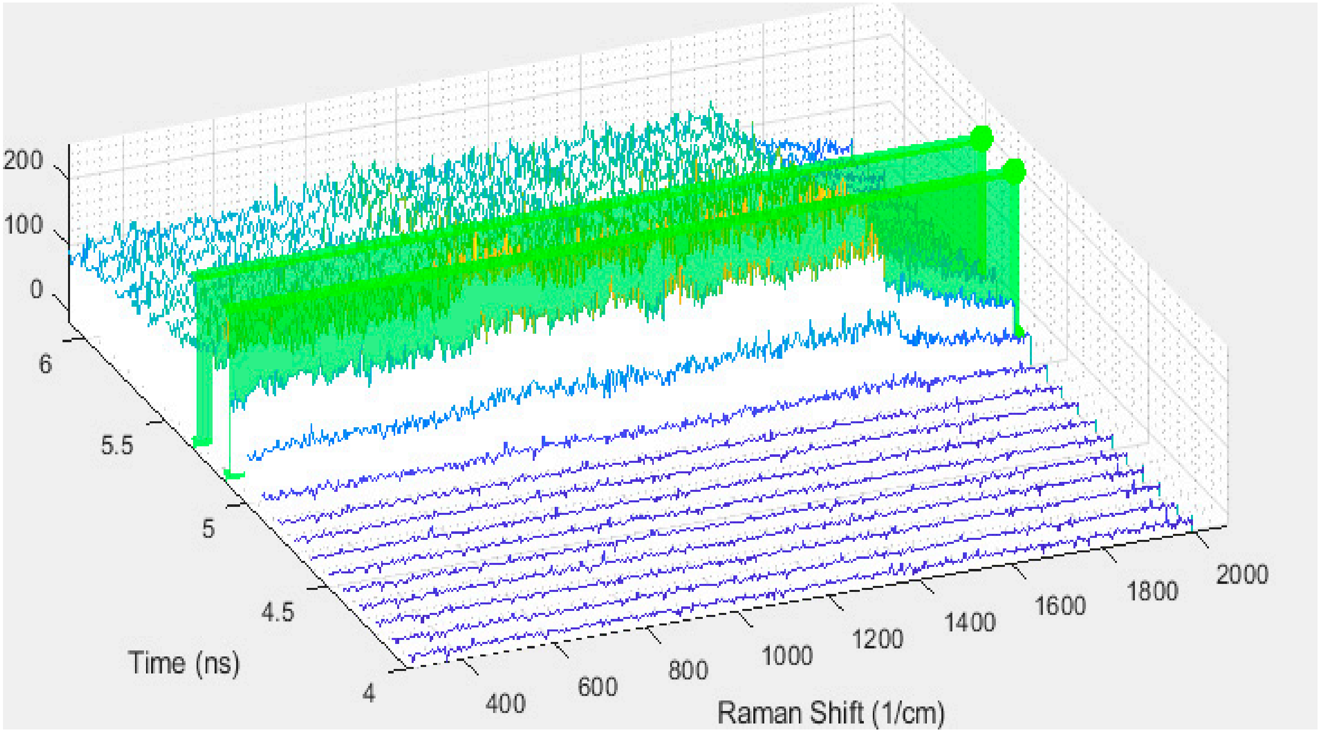

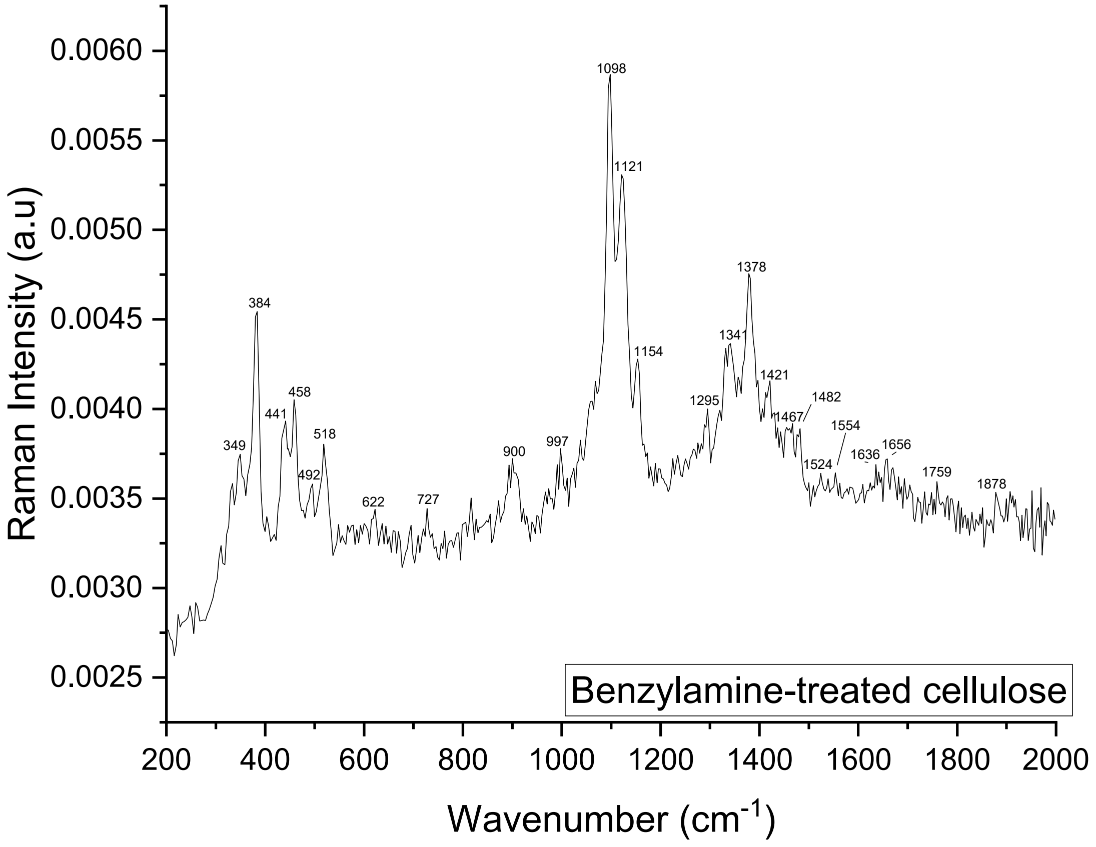

S19 Phenol-treated preactivated cotton fabrics

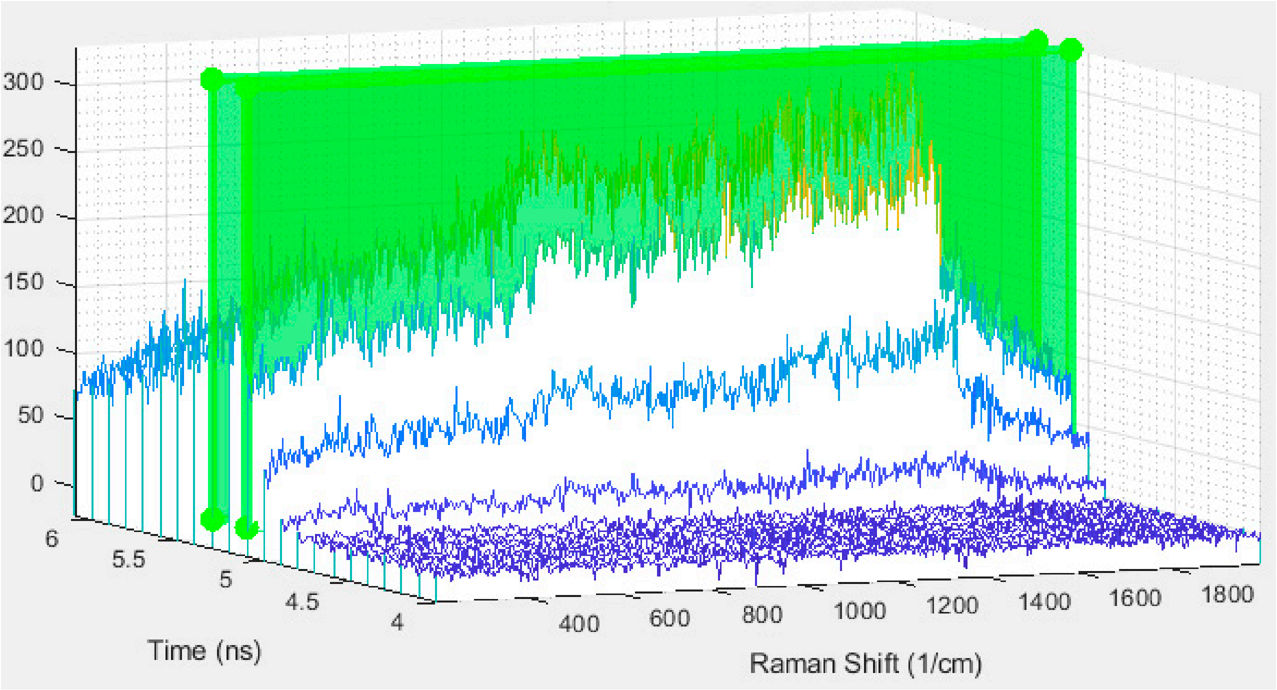

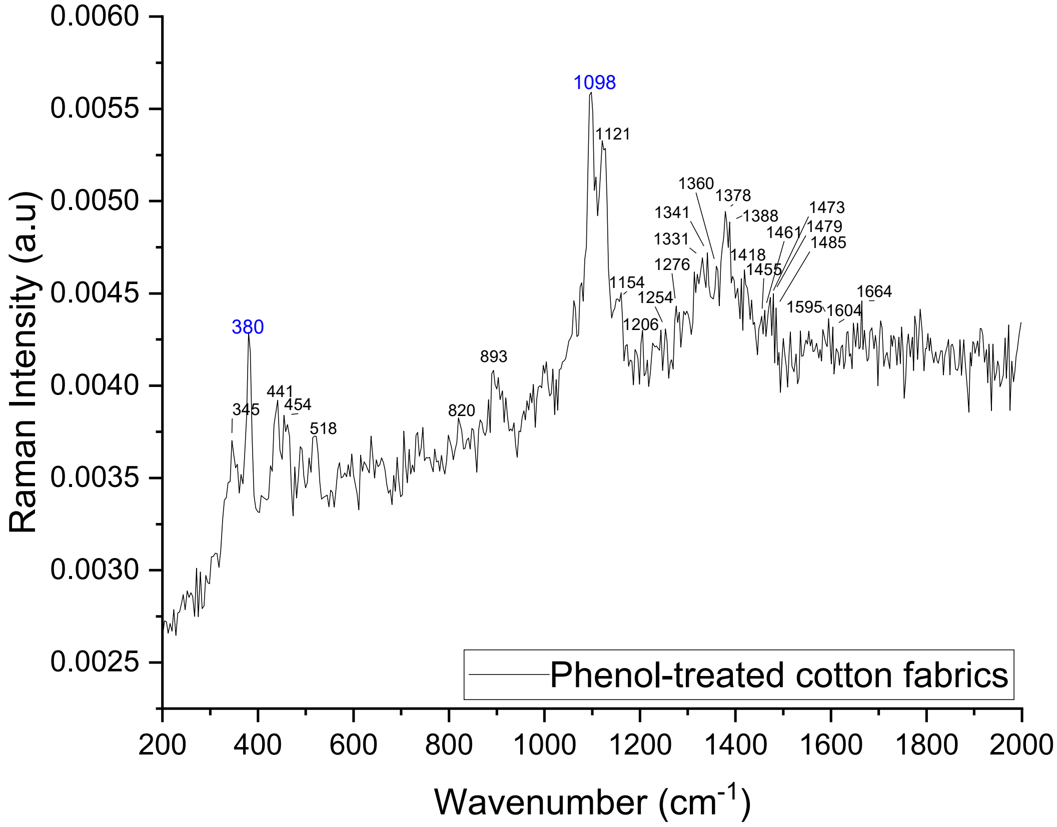

S20 Act-1-treated preactivated cotton fabrics

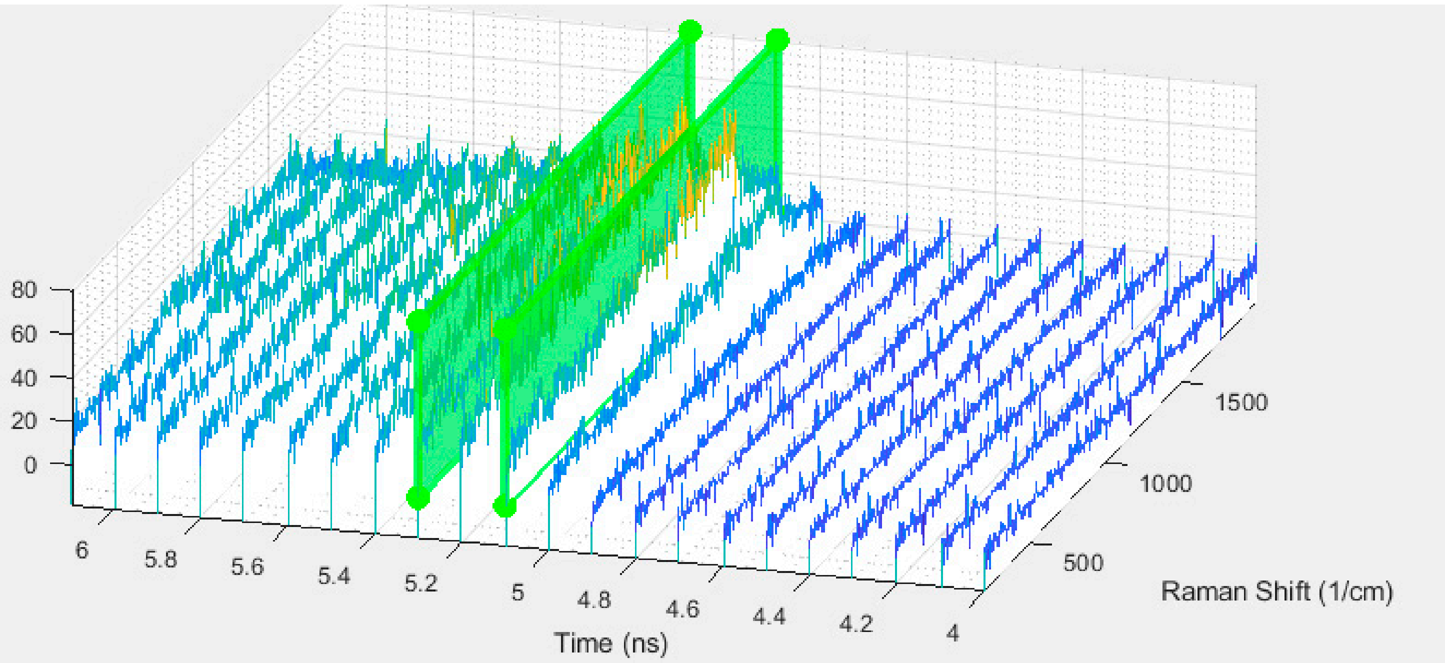

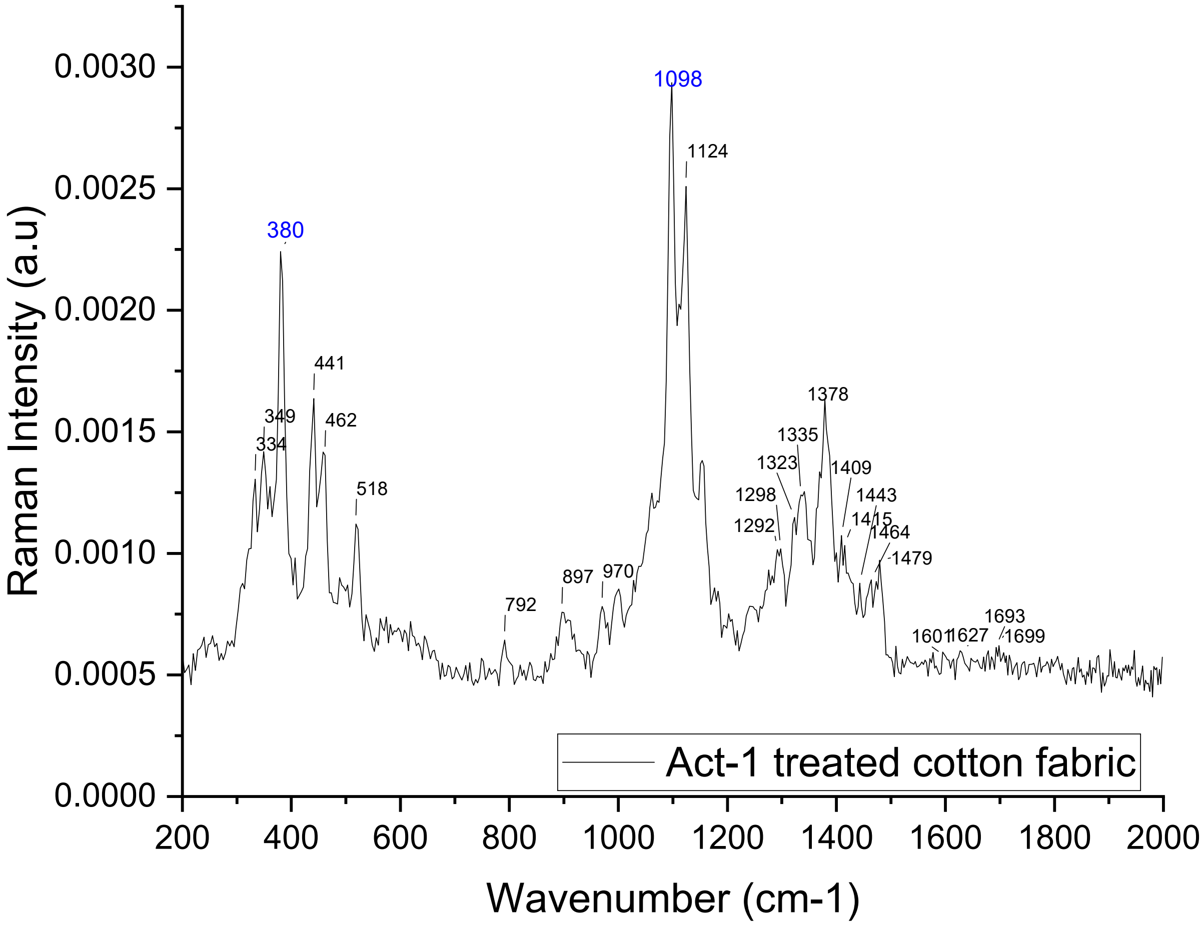

S21 Act-2-treated preactivated cotton fabrics

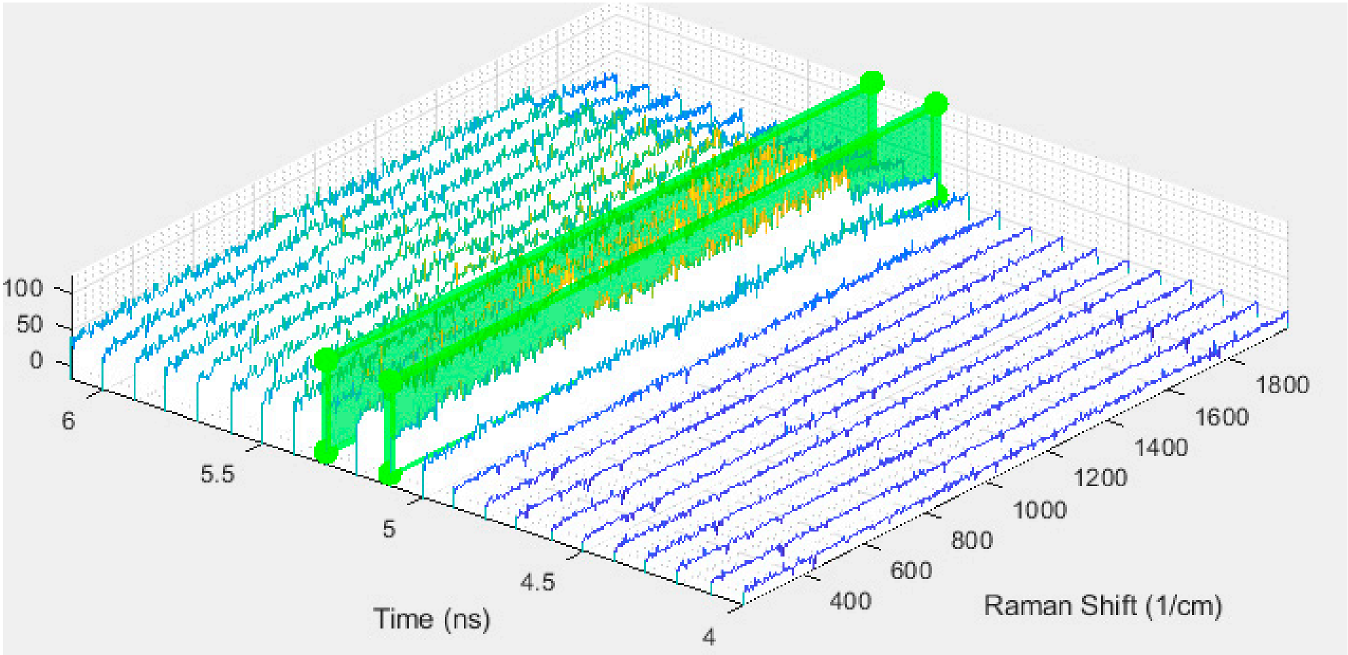

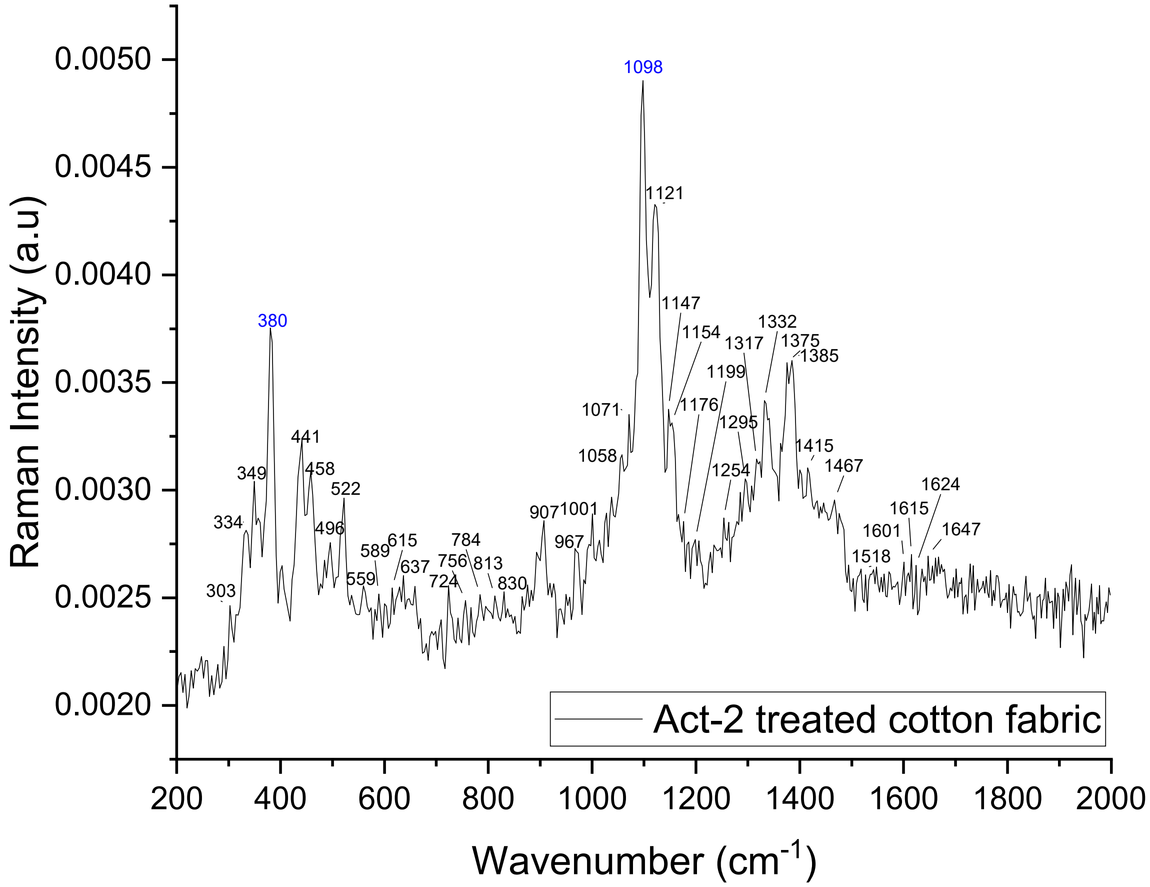

Supplement: Supplementary file 1 [file polymers-16-00149-s001.zip › polymers-2719910-supplementary.pdf]
